# Silencing P2Y_12_ and P2Y_13_ receptors rehabilitates the ADP-induced P2Y_1_-mediated osteogenic commitment of post-menopausal mesenchymal stromal cells

**DOI:** 10.1186/s12964-025-02355-0

**Published:** 2025-07-25

**Authors:** Catarina Bessa-Andrês, Rui Pinto-Cardoso, Maria Adelina Costa, Fátima Ferreirinha, José Marinhas, Rolando Freitas, Rui Lemos, Diogo Catelas, Adélio Vilaça, António Oliveira, Paulo Correia-de-Sá, José Bernardo Noronha-Matos

**Affiliations:** 1 Laboratório de Farmacologia e Neurobiologia, Instituto de Ciências Biomédicas Abel Salazar - Universidade do Porto (ICBAS-UP), Porto, 4050-313 Portugal; 2 Center for Drug Discovery and Innovative Medicines (MedInUP), Instituto de Ciências Biomédicas Abel Salazar - Universidade do Porto (ICBAS-UP), Porto, 4050-313 Portugal; 3 RISE-Health: Health Research Network, Instituto de Ciências Biomédicas Abel Salazar - Universidade do Porto (ICBAS-UP), Porto, 4050-313 Portugal; 4 Departamento de Química, Instituto de Ciências Biomédicas Abel Salazar - Universidade do Porto (ICBAS-UP), Porto, 4050-313 Portugal; 5 Serviço de Ortopedia e Traumatologia, ULS Gaia– Espinho, Vila Nova de Gaia, 4434-502 Portugal; 6 Serviço de Ortopedia, ULS de Santo António, Porto, 4099-001 Portugal; 7 Laboratório de Farmacologia e Neurobiologia / MedInUP / RISE-Health, Instituto de Ciências Biomédicas de Abel Salazar (ICBAS), Universidade do Porto (UP), Jorge Viterbo Ferreira Street, 228, Porto, 4050- 313 Portugal

**Keywords:** Mesenchymal stem cells, Post-menopausal osteogenesis, P2Y_1_ receptor, P2Y_12_ receptor, P2Y_13_ receptor, Cholesterol-rich lipid rafts/Caveola

## Abstract

**Background:**

Participation of ADP-sensitive metabotropic P2Y_1_, P2Y_12_ and P2Y_13_ receptors in human osteogenesis is controversial. Here, we investigated the variations in the expression and bone-forming properties of the P2Y_1_R in osteogenic-differentiating bone marrow-derived mesenchymal stromal cells (BM-MSCs) isolated from post-menopausal (Pm) women. We also tested whether observed P2Y_1_-related functional deficits result from the crosstalk with co-localized P2Y_12_ and P2Y_13_ receptors.

**Methods:**

Pm BM-MSCs were cultured in an osteogenic-inducing medium in either the absence or presence of the selective P2Y_1_ receptor agonist, MR2365; this compound was applied alone or after cells’ incubation with selective P2Y_12_ and P2Y_13_ receptor antagonists or short hairpin RNAs designed to silence P2Y_12_ or P2Y_13_ receptors gene expression.

**Results:**

BM-MSCs present immunoreactivity against all ADP-sensitive P2Y receptor subtypes, but their relative density varied among different Pm women and with the time of the cells in the culture. The P2Y_1_receptor agonist increased the alkaline phosphatase activity and bone nodule formation in BM-MSCs originating from a younger female, but it failed to promote the osteogenic differentiation of BM-MSCs from Pm women unless P2Y_12_ or P2Y_13_ receptors are blocked with AR-C66096 and MRS211, respectively. Silencing the P2Y_13_, but not the P2Y_12_, receptor gene expression restored the P2Y_1_-mediated osteogenic commitment of Pm BM-MSCs. The P2Y_1_ receptor agonist failed to elicit [Ca^2+^]_i_ transients inside Pm BM-MSCs except after acute cholesterol depletion and lipid rafts disruption with methyl-β-cyclodextrin to prevent the P2Y_1_/P2Y_13_ receptors interplay.

**Conclusions:**

Thus, personalized offsetting the activity and/or expression of P2Y_13_ receptor (and P2Y_12_) may be a good strategy to rehabilitate the P2Y_1_-mediated osteogenic potential of BM-MSCs and to reduce the fracture risk in Pm women.

**Supplementary Information:**

The online version contains supplementary material available at 10.1186/s12964-025-02355-0.

## Graphical abstract

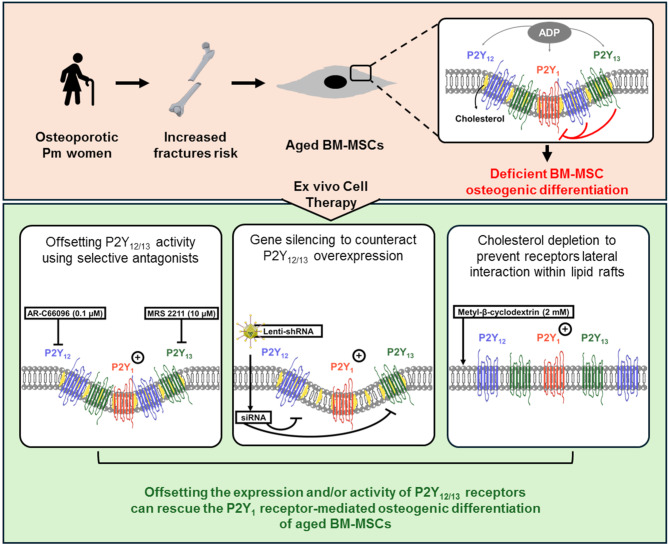

## Background

Declining skeletal strength and increased risk of falling dramatically increase the fracture risk with advancing age. The most serious fractures of the wrist, hip, pelvis and spine are associated with primary osteoporosis, a metabolic bone disorder that is characterized by a progressive decline of bone mass and microarchitecture deterioration [1]. Age- and sex-dependent bone loss and osteoporotic fractures are highly prevalent in post-menopausal (Pm) women; this constitutes a major burden for health care systems due to increased morbidity, excess mortality, decreased quality of life and loss of autonomy in this growing segment of the global population. Considering this scenario, advances in risk assessment, and non-pharmacologic and pharmacologic management of Pm bone mass loss and osteoporosis warrant constant updates. While current drug therapies (e.g. bisphosphonates, teriparatide, denosumab) against primary osteoporosis outweigh the risks, the long-term use of these drugs may lead to complications [2, 3]. This fully justifies the need for a better understanding of this disease condition, which may be valuable for prompting adequate risk assessment and innovative molecular drug targets and cell-based therapies to rebalance bone homeostasis.

Mesenchymal stromal cells (MSCs) hold great promise in tissue engineering to replace tissue damage, promote regeneration, and control immune-mediated diseases [4] since they (i) are multipotent (differentiate into osteoblasts, chondrocytes and adipocytes), (ii) self-renewable, (iii) have high proliferative capability in the undifferentiated state, (iv) release trophic factors, and (v) have immunosuppressive activity [5–7]. These findings explain the exponentially increasing popularity of MSCs in clinical trials since 2004 [7], some of those including the management of critical bone defects and fracture mal-union (see e.g. NIH ClinicalTrials.gov and [8, 9]). Nevertheless, the ageing of MSCs limits their ability to differentiate into bone-forming cells; thus, targeting age-associated alterations in MSCs to restore their osteogenic commitment may be an appealing strategy to promote bone repair and to increase their clinical usage in tissue bioengineering, as previously anticipated by our research group [10].

Adenine and uracil nucleotides belong to a group of constitutively released signalling molecules playing important autocrine/paracrine roles as local regulators of bone homeostasis and remodelling via the activation of ionotropic P2X and metabotropic P2Y purinoceptors [10, 11]. Huge amounts of purines and pyrimidines are released to the bone microenvironment upon mechanical stimulation of MSC-derived osteoprogenitors and mature osteoblasts [reviewed in 10]. Once released from cells, ATP and UTP can be dephosphorylated into other biologically active products, like ADP, UDP and adenosine, by a cascade of membrane-bound NTPDases [12, 13]. Our group hypothesized that targeting the “purinome”, comprising nucleotide-releasing sites, ecto-NTPDases and purinoceptors, may orchestrate to restore the osteogenic differentiation and bone repair potential of aged MSCs from Pm women [10]. This endeavour is achieved by either activating metabotropic P2Y_6_ or ionotropic P2X7 receptors with selective UDP and ATP stable analogues, respectively [14, 15]. Alternatively, silencing the NTPDase3 overshooting activity in bone marrow-derived MSCs (BM-MSCs) from Pm women may also rehabilitate their osteogenic commitment using either lenti-shRNAs, the hN3-B3_S_ monoclonal antibody, or anthraquinone enzyme inhibitors, like PSB 06126 [16].

Concerning the osteogenic role of ATP metabolites in human MSCs, only adenosine has deserved sufficient attention [e.g. 17]. Data showed that the most abundant adenosine A_2B_ receptor consistently promotes the osteogenic differentiation of BM-MSCs of Pm women, but this effect is balanced by the tonic activation of co-located A_1_ or A_2A_ receptors determining whether the cells are driven into proliferation or differentiation. In the literature, few and conflicting reports still exists regarding the role of ADP-sensitive plasma membrane-bound purinoceptors, namely P2Y_1_, P2Y_12_ and P2Y_13_, in human osteogenesis. The P2Y_1_ receptor participates in the responsiveness of bone cells to parathyroid hormone (PTH) eventually resulting in bone growth [18, 19]. Nevertheless, the expression and function of P2Y_1_ receptors are highly heterogeneous among human bone donors and with the differentiation stage of osteoblasts in primary cultures; the results are more consistent when using immortalized osteoblast-like cells, like those originated from the osteosarcoma cell line SaOS-2 [20]. Regarding the P2Y_12_ receptor, prolonged application of low micromolar concentrations of the P2Y_12_ receptor antagonist, clopidogrel, reduced cell viability/proliferation, alkaline phosphatase (ALP) activity, collagen production, and bone nodule formation in rat osteoblastic cell cultures. Used in a dose of 1 mg/kg/day for 4 weeks, this antiplatelet medication reduced the trabecular number and volume of long bones and spine in adult ovariectomized mice [21]. Surprisingly, the clopidogrel-induced bone loss was also detected in mice lacking the P2Y_12_ receptor [22]. On the other hand, clopidogrel administered to a cohort of Danish patients followed up for 10 years revealed a puzzling biphasic drug relationship concerning the risk of fractures, where low doses may have protective effects, whereas prolonged or high-dose exposure seemed to impair bone integrity [23]. Such non-linear responses suggest complex regulation of purinergic signalling in bone homeostasis, further supporting the need to understand individual receptor contributions, as aimed in this study. Unfortunately, the role played by the P2Y_13_ receptor in MSCs differentiation into osteoblast cell lineage, while inhibiting adipogenesis, has only been reported in rodents. Data showing that P2Y_13_ receptor knockout mice had reduced bone formation support this theory [24–26]. Regarding ADP-sensitive P2Y receptors signalling pathways it is well established that the P2Y_1_ receptor typically couples via a G_q/11_ protein anchor to the phospholipase C/IP_3_/diacylglycerol (PLC/IP_3_/DAG) pathway. In contrast, P2Y_12_ and P2Y_13_ receptors usually inhibit adenylate cyclase activity by coupling to G_i/o_ proteins [27]. However, some studies have shown that the P2Y_13_ receptor can also activate the PLC pathway [28–32], whereas there is no evidence of this for the P2Y_12_ receptor. Interestingly, the P2Y_12_ receptor is also able to promote the P2Y_1_-dependent calcium response [33].

Several hypothesis might contribute to explain the aforementioned conflicting results in the literature about the role of ADP-sensitive purinoceptors, P2Y_1_, P2Y_12_ and P2Y_13_, in the remodelling human bone. These include (i) heterogeneity in P2Y_1_, P2Y_12_ and P2Y_13_ receptors distribution/density, activity and crosstalk within the plasma membrane among osteoprogenitor cells *vis a vis* differentiated osteoblasts and osteoblast-like cell lines [34]; (ii) age-related changes in nucleotides-induced differentiation of MSC-derived progenitors into bone-forming cells [10]; and (iii) existence of striking species differences in the molecular composition and activity of ADP-sensitive receptors, particularly between humans and rodents [35]. This prompted us to investigate variations in the expression and bone-forming capacity of the P2Y_1_ receptor subtype in osteogenic-differentiating primary cultures of BM-MSCs from Pm women. We also evaluated whether the expression density and functional crosstalk with co-localized ADP-sensitive receptors, namely the P2Y_12_ and P2Y_13_, could provide explanations for the inconsistencies in cell distribution and osteogenic function of the P2Y_1_ receptor among different individuals.

## Methods

### Reagents, lenti‑shRNAs and antibodies

3,4-dihydroxy-9,10-dioxo-2 anthracenesulfonic acid sodium salt (Alizarin red S), 3-[4,5-dimethylthiazol-2-yl]-2,5-diphenyltetrazolium bromide (MTT), methyl-β-cyclodextrin (MβC), phosphate-buffered saline solution (PBS), *p*-nitrophenyl phosphate, trypsin, type I collagenase and cell culture reagents were obtained from Sigma-Aldrich (St. Louis, MO, USA; RRID: SCR_008988). 2-(propylthio)adenosine-5’-*O*-(β,γ-difluoro methylene)triphosphate tetrasodium salt (AR-C66096), 2-[(2-chloro-5-nitrophenyl)azo]-5-hydroxy-6-methyl-3-[(phosphonooxy)methyl]-4-pyridinecarboxaldehyde disodium salt (MRS 2211), 2’-deoxy-N^6^-methyladenosine 3’,5’-bisphosphate tetrasodium salt (MRS 2179), and [[(1*R*,2*R*,3*S*,4*R*,5*S*)-4-[6-amino-2-(methylthio)-9*H*-purin-9-yl]-2,3-dihydroxybicyclo[3.1.0]hex-1-yl]methyl] diphosphoric acid mono ester trisodium salt (MRS 2365) were obtained from Tocris Bioscience (Bristol, UK; RRID: SCR_003689). All primary anti-human and secondary conjugated antibodies used in this study have been previously validated [36]. Anti-P2Y_1_ (anti-rabbit, Cat# APR-009, RRID: AB_2040070), anti-P2Y_12_ (anti-rabbit, Cat No. APR-012, RRID: AB_2040074) and anti-P2Y_13_ (anti-rabbit, Cat No. APR-017, RRID: AB_2040076) were from Alomone Labs (Jerusalem, Israel; RRID: SCR_013570); secondary antibodies Alexa Fluor 488-labelled (anti-rabbit, Cat No. A21206, RRID: AB_2535792) and Alexa Fluor 568-labelled (anti-rabbit, Cat No. A10042, RRID: AB_2534017), the fluorescent calcium indicator Fluo-4 NW, and the FM4-64 dye were supplied by Molecular Probes (Invitrogen, Carlsbad, CA, USA; RRID: SCR_013318). pGFP-C-shLenti Vectors were supplied from ORIGENE (Rockville, MD, USA; RRID: SCR_008985).

### Isolation, culture and osteogenic differentiation of human BM-MSCs

Bone marrow samples were harvested from the neck of the femur of eighteen Pm women (73 ± 5 years old) undergoing total hip replacement due to non-inflammatory degenerative osteoarthrosis. For comparison, we also used bone marrow from the lamina and articular processes of vertebrae of a 37-year-old female, who required bone engraftment for spinal fusion to correct low back pain with spinal instability. The procedures for handling bone marrow specimens and culturing adherent cells followed previous reports from our group [16, 37]. The phenotypic characterization of isolated human BM-MSCs concerning steaminess and tri-lineage differentiation capability has been previously documented in plastic-adherent cells by our group using flow cytometry (e.g. CD105pos/CD29pos/ CD117pos vs. CD14neg/CD45neg) [15] and culture differentiation assays [38]. Only first subcultures were used in this study to minimize the impact of in vitro cell senescence and phenotypic modifications. BM-MSCs were seeded at 2.5 × 10^4^ cells/mL density and cultured for 35 days in α-minimal essential medium (α-MEM)-based osteogenic-inducing medium supplemented with 10% foetal bovine serum (FBS), 100 U/mL penicillin, 100 µg/mL streptomycin and 2.5 µg/mL amphotericin B, along with 50 µg/mL ascorbic acid, 10 mM β-glycerophosphate and 10 nM dexamethasone. Under these experimental conditions, high expression levels of ecto-5′-nucleotidase/CD73 anticipate that these cells are highly committed to osteogenesis [16, 37]. All experimental protocols used individualized samples with no pooling from different individuals at any circumstance.

### P2Y_12_ and P2Y_13_ gene silencing using a lentivirus‑coupled shRNA

P2Y_12_ and P2Y_13_ gene silencing was achieved by exposing the BM-MSCs to pGFP-C-shLenti Vectors, respectively, code TL302719V and TL302718V (ORIGENE, Rockville, MD, USA; RRID: SCR_008985). Four specific sequences were employed for each receptor. For P2Y_12_, the sequences used were: TL302719VA (TGACCAACAGGCAGCCGAGAGACAAGAAT), TL302719VB (GTTGGACTTATCACAAATGGCCTGGCGAT), TL302719VC (AGAGTTCGGTCTAGTCTGGCATGAAATAG) and TL302719VD (TCTCTTTGCCTAACATGATTCTGACCAAC). For P2Y_13_, the used sequences were: TL302718VA (TGAACACCACAGTGATGCAAGGCTTCAAC), TL302718VB (CCTGCCAAATACGATCTTGAGCAACAAGG), TL302718VC (GCCGACTTGATAATGACACTCATGCTTCC) and TL302718VD (GGCTCATAGCCTTTGACAGATTCCTCAAG). We used a mismatch scramble sequence (TR30021V) as negative control. The cells were incubated with the lenti-shRNAs for 24 h, using half the volume of the standard cell medium to enhance transduction efficiency. The same protocol was applied concerning the 4 specific sequences, the scrambled sequence, and non-treated control cultures [39]. Validation experiments were performed varying the multiplicities of infection (MOI) from 1, 3 to 10 (see Supplementary Figs. 1 and 2) [39]. TL302719VA at MOI 3 and TL302718VD at MOI 1 were the most effective sequences for silencing P2Y_12_ and P2Y_13_ gene expression, respectively. Criteria to select the optimal experimental conditions were as follows. First, significant reductions in receptors density. Second, absence of effect of the scramble sequence on receptors density (see Supplementary Fig. 3a) and osteogenic differentiation parameters assessed by measuring the ALP activity and bone nodule formation (see Supplementary Fig. 3c and 3d, respectively). Third, absence of significant (*P* > 0.05) changes concerning cells viability between scramble- and shRNA-treated cells (MTT assay; see Supplementary Fig. 3b).

### Characterization of BM-MSCs growth and osteogenic differentiation

The MTT assay was used to assess cells viability/proliferation, as previously described [14–17]. Increases in alkaline phosphatase (ALP) activity in the cultures are an indication of the osteogenic differentiation of BM-MSCs. ALP activity was measured in cell lysates using a colorimetric assay based on the hydrolysis of *p*-nitrophenyl phosphate (PNP), as established previously [14–16, 37]. Calcium mineralization of bone nodules was detected by the Alizarin Red staining on culture day 35 using a microscope (Olympus CKX41, RRID: SCR_023725; Tokyo, Japan; RRID: SCR_017564) equipped with a digital camera (Olympus SC30, Tokyo, Japan; RRID: SCR_017564) running the Olympus Analysis GetIT 5.1 software (Tokyo, Japan; RRID: SCR_017564). After size calibration, captured images were analysed with the Image J 1.37c software (RRID: SCR_003070; NIH, Bethesda, MD, USA) for quantification of total bone-nodule areas [14, 16, 37].

### Immunofluorescence staining and confocal microscopy observation of osteogenic differentiating BM-MSCs

For immunofluorescence staining, paraformaldehyde fixated BM-MSCs grown for 7 to 21 days in glass-bottom chamber slices were incubated for 2 h in the dark with the following primary antibodies: rabbit anti-human P2Y_1_ (1:50, Cat# APR-009, RRID: AB_2040070), rabbit anti-human P2Y_12_ (1:100, Cat# APR-012, RRID: AB_2040074) and rabbit anti-human P2Y_13_ (1:25, Cat# APR-017, RRID: AB_2040076). As secondary antibodies, we used the Alexa Fluor 488 (1:1500, anti-rabbit, Cat. No. A21206, RRID: AB_2535792) or the Alexa Fluor 568 (1:1500, anti-rabbit, Cat. No. A10042, RRID: AB_2534017) for 1 h in the dark. The slides were mounted with VectaShield containing DAPI. Observations were carried out using a laser-scanning spectral confocal microscope (Olympus FV1000, RRID: SCR_020337; Tokyo, Japan; RRID: SCR_017564) running the Fluoview FV1000 Advanced Software (4.0.3.4 version, RRID: SCR_014215; Olympus, Tokyo, Japan; RRID: SCR_017564) for image acquisition and data analysis. We performed negative controls to detect non-specific fluorescence by omitting the primary antibodies (see Supplementary Fig. 4a). Regions of interest (ROIs) representing individual cells were manually outlined, and the average pixel intensity inside individual cells determined in each image. The background fluorescence outside cells was subtracted from all analysed ROIs. For further details on image acquisition and data analysis, refer to previous publications of our group [16, 37].

### Fluorescent [Ca^2+^]_i_ oscillations in BM-MSCs undergoing osteogenic differentiation

Intracellular Ca^2+^ ([Ca^2+^]_i_) oscillations were monitored in osteogenic differentiating BM-MSC populations loaded with the cell-permeant fluorescent calcium indicator, Fluo-4NW (Invitrogen, Carlsbad, CA, USA; RRID: SCR_013318) using a multi-detection microplate reader (Synergy HT, RRID: SCR_020536; BioTek Instruments, Vermont, USA) running the BioTek’s Gen5™ Data Analysis Software (RRID: SCR_017317; BioTek Instruments, Vermont, USA) [40]. On the day of the experiment, the cells were washed twice with warm (37 °C) gassed (95% O_2_ + 5% CO_2_) Tyrode’s solution containing (mM): NaCl 137, KCl 2.7, CaCl_2_ 1.8, MgCl_2_ 1, NaH_2_PO_4_ 0.4, NaHCO_3_ 11.9 and glucose 11.2, at pH 7.4. After loading the cells with Fluo-4NW (2.5 µM, in 1× HBSS, 20 mM HEPES and 2.5 mM probenecid) for 30 min, the 96-well plates were transferred into the microplate reader to monitor fluorescent [Ca^2+^]_i_ oscillations per well in the time-lapse mode. The temperature inside the microplate reader was kept at 32 °C to preserve cells integrity during recordings. The cells were exposed intermittently (once every five seconds for about 30 min) to fluorescent light using a tungsten halogen lamp; excitation filtered at 485/20 nm and emission recorded at 528/20 nm. [Ca^2+^]_i_ oscillations were calibrated to the maximal [Ca^2+^]_i_ load inside cells determined by incubation with ionomycin (5 µM, 100% response) at the end of the experiment [36, 40].

For single-cell [Ca^2+^]_i_ imaging we used a laser-scanning confocal microscope (Olympus FV1000, RRID: SCR_020337; Tokyo, Japan; RRID: SCR_017564) in the time-lapse mode, as previously described [15]. After loading the cells with the fluorescent calcium dye, Fluo-4NW, as mentioned above, cell culture dishes were mounted on a temperature-controlled perfusion chamber (kept at 32 °C) placed on the stage of an inverted laser-scanning confocal microscope equipped with a 20x magnification objective lens (LUCPLFLN 20x PH; NA: 0.45) (Olympus, Tokyo, Japan; RRID: SCR_017564). The cells were continuously superfused (1 mL/min) with gassed (95% O_2_ + 5% CO_2_) Tyrode’s solution (the same composition as above) using a Peri-Star Pro 4-channel peristaltic pump (World Precision Instruments; Hitchin, Hertfordshire, UK; RRID: SCR_008593). Dye excitation was performed using the 488 nm Multi-line Ar laser; fluorescence emission was recorded through a 510–560 nm band pass filter placed in the detection path of the confocal microscope. Time-lapse sequences were captured each every 20 s for about 30 min. Digitized images were processed offline. Regions of interest were outlined as bright areas with minimal background noise. [Ca^2+^]_i_ signals were calibrated by the maximal [Ca^2+^]_i_ load inside each cell using ionomycin (5 µM, 100% response), as previously described above [36, 40].

In some experiments, we monitored single-cell [Ca^2+^]_i_ oscillations in parallel to the incorporation of FM4-64™ (5 µM) (Invitrogen, Carlsbad, CA, USA; RRID: SCR_013318), a styryl fluorescent dye used to assess real-time membrane plasticity changes in living cells [41]. To this end, although we used the 488 nm Multi-line Ar laser to excite both dyes, fluorescence emission of the FM4-64™ was detected separately at 665–765 nm.

Intracellular [Ca^2+^]_i_ signals were triggered using increasing concentrations of the selective P2Y_1_ receptor agonist, MRS 2365 (10 nM-10 µM), which was applied either alone or in the presence of selective P2Y_12_ or P2Y_13_ receptor antagonists, AR-C66096 (0.1 µM) and MRS 2211 (10 µM), respectively. The role of acute depletion of cholesterol from the plasma membrane, which results in the perturbation of lipid rafts architecture eventually leading to the disappearance of metabotropic receptors-enriched caveola membrane microdomains, was assessed using methyl-β-cyclodextrin (MβC, 2 mM) instead of P2Y receptor antagonists [42].

### Ethics approval and humans consent to participate

Informed consent to use the biological material that would be otherwise discarded was obtained. All procedures were approved within the scope of the project “BONE-PURI(NO)AGEING– Regeneration of aged human bone through purinome activation in mesenchymal stem cells– preclinical studies”, followed by the project “BONEREGENERA - New insights of the purinome in predicting bone healing by mesenchymal stromal cells in osteoporotic patients” by the Ethics Committees of Centro Hospitalar de Vila Nova de Gaia– Espinho (registration nº 137/2018-2, endorsed on January 10, 2019) and of Gabinete Coordenador de Investigação / DEFI– Centro Hospitalar Universitário de Santo António (CHUdSA, registration nº 2021-002(001-DEFI-001-CE, endorsed on September 01, 2021), and of Instituto de Ciências Biomédicas Abel Salazar (Medical School) of the University of Porto. The investigation conforms to the principles outlined in the Declaration of Helsinki.

### Presentation of data and statistical analysis

Data are presented as scatter dot plots (with mean ± SD) bar charts or symbols representing mean ± SD for an *n* number of individuals. Each experimental condition was tested using at least 6 replicates per individual. No prior sample size calculation was conducted. Concentration-response curves were generated by fitting data using a non-linear regression function: log(drug) versus response. It was assumed that data share common best-fit values for both top and bottom, with a hill slope set to 1. The fitting process utilized the least squares method. Due to restricted access to similar human samples and a limited pool of initial cell density, we were unable to perform all the indicated assays in all collected human samples. We assessed data normality using the D’Agostino & Pearson or Shapiro-Wilk tests, depending on sample size. Based on the normality test results, statistical analyses included parametric tests (ordinary one-way ANOVA with uncorrected Fisher’s LSD test, RM one-way ANOVA with the Geisser-Greenhouse correction and uncorrected Fisher’s LSD test, unpaired t-test and multiple unpaired t-tests) or non-parametric tests (two-tailed Mann Whitney test and Kruskal-Wallis with or without Dunn’s multiple comparison test). A significance threshold of *P* < 0.05 (95% confidence interval) was used to determine statistically significant differences. Data analysis was performed using the Prism 10.0.2 TM software (RRID: SCR_002798; GraphPad Software, CA, United States).

## Results

### Relative density of ADP-sensitive P2Y_1_, P2Y_12_ and P2Y_13_ receptors in osteogenic differentiating BM-MSCs of Pm women vs. a younger control

**Fig. 1:**
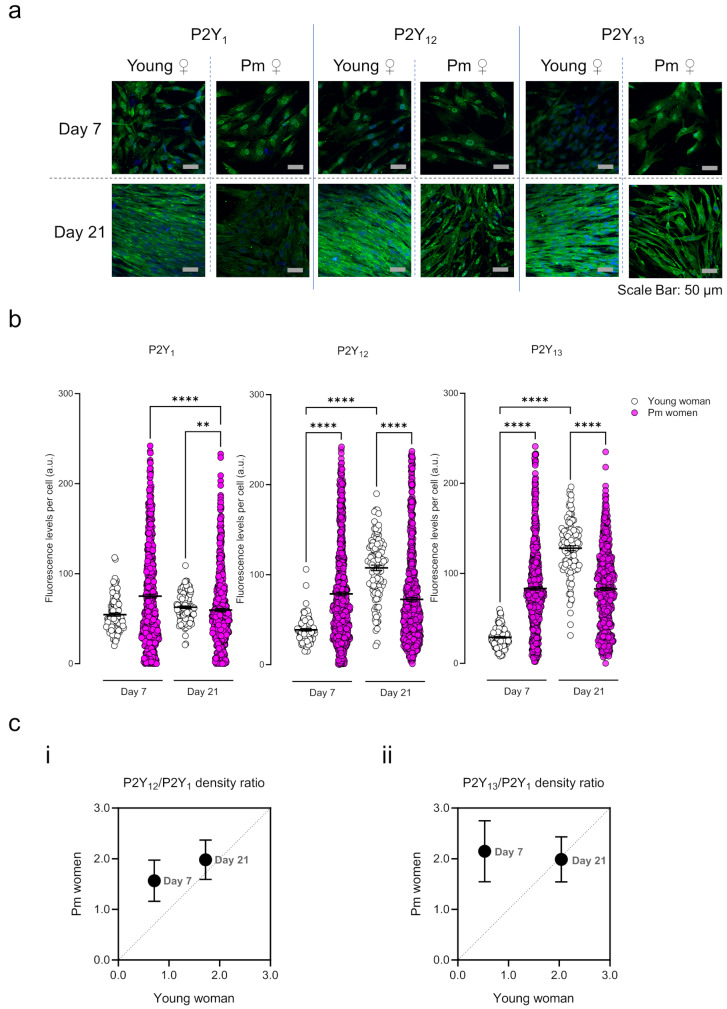
Pattern of ADP-sensitive P2Y receptors immunoreactivity in cultured BM-MSCs from young and Pm women. (**Panel a**) presents representative immunofluorescence confocal micrographs of BM-MSCs from a young female (37 years old) and a Pm woman (67 years old) grown for 7 and 21 days in an osteogenic-inducing medium stained against P2Y_1_, P2Y_12_ and P2Y_13_ receptors (green). Blue dots represent nuclei stained with DAPI. The scale bar is 50 μm. In (**panel b**), ordinates represent the fluorescence intensity per cell (arbitrary units, a.u.) of the indicated immunotarget as a function of the number of days in culture (days 7 and 21). Scatter dot plots (with mean ± SD) represent pooled data from a total of 105–120 cells analysed from 1 young woman (37 years old) and a total of 584–708 cells analysed from 6 Pm women (73 ± 6 years old). **P < 0.01 and ****P < 0.0001 (non-parametric Kruskal-Wallis with Dunn’s multiple comparison test) represent significant differences. Data in **panel c)** shows the (i) P2Y_12_/P2Y_1_ and (ii) P2Y_13_/P2Y_1_ density ratios at culture day 7 and 21. Symbols (mean ± SD) represent pooled data from 9 Pm women (73 ± 5 years old) and 1 young female (37 years old). The dashed horizontal line represents the identity between young and Pm women ratios

Figure 1a shows that BM-MSCs undergoing osteogenic differentiation exhibit immunoreactivity against all ADP-sensitive P2Y receptors, but the relative density of P2Y_1_, P2Y_12_ and P2Y_13_ subtypes differ with age (Pm women vs. younger female) and with the time (day 7 vs. day 21) of the cells in culture. The P2Y_1_ receptor density declined with time (7 > 21 days) in BM-MSCs from Pm women, whereas P2Y_12_ and P2Y_13_ receptor subtypes remained fairly constant throughout the culture period (Fig. 1b). This pattern contrasts with that obtained using BM-MSCs isolated from a 37-years old female; while the P2Y_1_ receptor density was fairly conserved throughout the culture period, the immunoreactivity against the P2Y_12_ and P2Y_13_ receptor subtypes increased from culture day 7 to 21 by 3- and 4-fold, respectively (Fig. 1b). Thus, the relative abundance of ADP-sensitive P2Y receptors in the younger female changed from P2Y_1_ > P2Y_12_ > P2Y_13_ to P2Y_13_ > P2Y_12_ > > P2Y_1_ from culture day 7 to 21, but no such difference was observed in the cells originated from Pm women (Fig. 1b). Figure 1c shows that Pm women overexpress P2Y_12_ and P2Y_13_ compared with the P2Y_1_ receptor subtype at early culture stages (day 7), but the osteogenic differentiation of BM-MSCs in culture [see, e.g. Ref. 14–16] attenuates the differences of the P2Y_12_/P2Y_1_ and P2Y_13_/P2Y_1_ density ratios between Pm women and the younger control (Fig. 1c).

### Blockage of the P2Y_1_ receptor tonus decreases the osteogenic differentiation of BM-MSCs in the younger female, but not in Pm women, while the opposite arises by blocking the P2Y_12_ and P2Y_13_ receptor subtypes

**Fig. 2:**
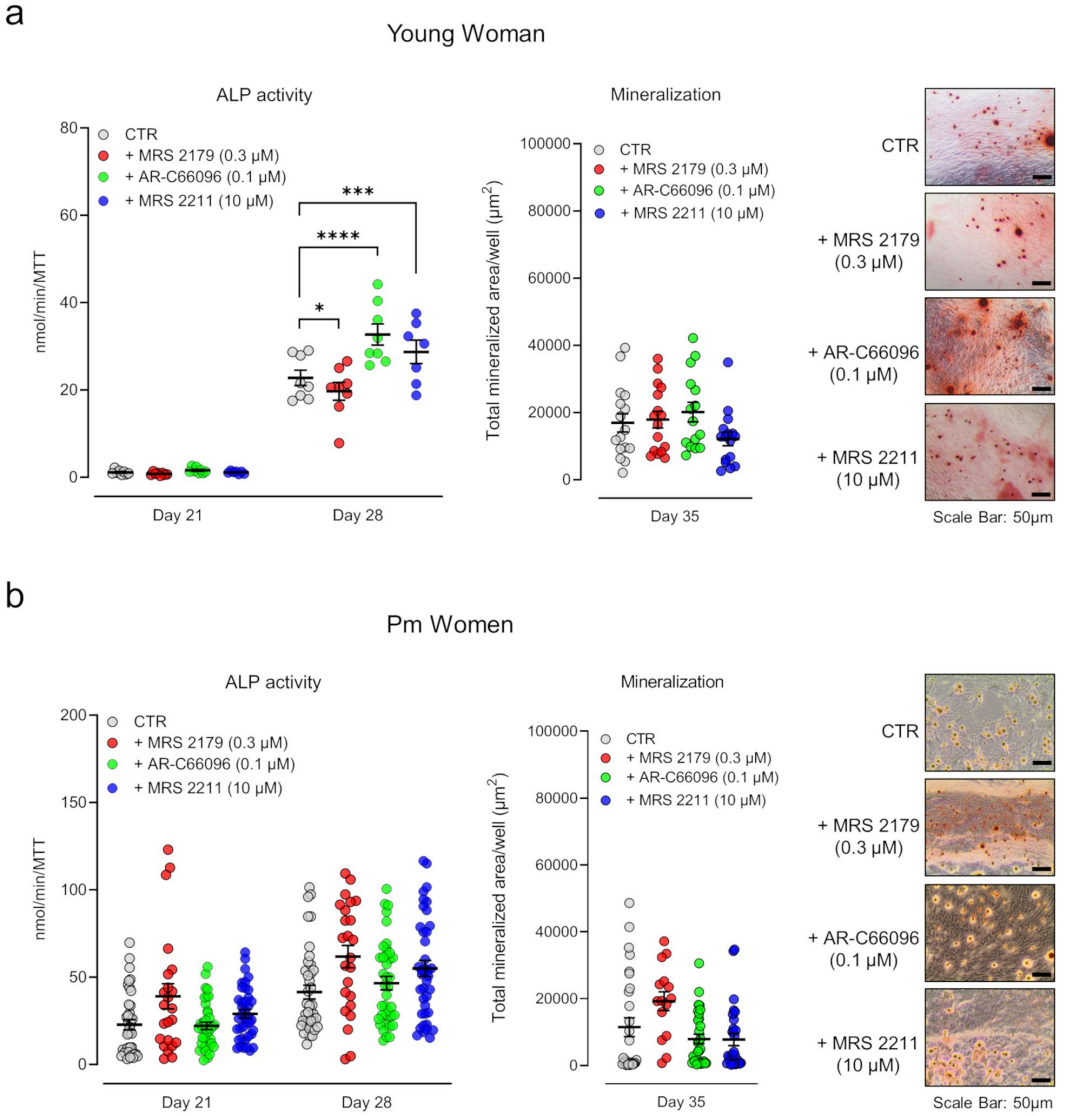
Tonic activation of ADP-sensitive P2Y receptors is impaired in Pm BM-MSCs. BM-MSCs were allowed to grow in an osteoblast-inducing medium for 21, 28 and 35 days, either in the absence or presence of selective P2Y_1_ (MRS 2179, 0.3 µM), P2Y_12_ (AR-C66096, 0.1 µM) and P2Y_13_ (MRS 2211, 10 µM) receptor antagonists. **Panels (a) and (b)** show, for (**a**) young and (**b**) Pm women BM-MSCs, the ALP activity (in nmol/min/MTT) and extracellular matrix mineralization (in µm^2^). Scatter dot plots (with mean ± SD) represent pooled data from 1 young woman (37 years old) and 2 to 5 Pm women (74 ± 5 years old); four to sixteen replicates were performed per individual. *P < 0.05, ***P < 0.001 and ****P < 0.0001 (parametric ordinary one-way ANOVA with uncorrected Fisher’s LSD test for young woman results and non-parametric Kruskal-Wallis test with uncorrected Dunn’s test for Pm women) represent significant differences. Right hand-side images show typical micrographs displaying bone nodule formation (red-brownish spots) in BM-MSC cultures from a young female (37 years old) and a Pm woman (71 years old). Scale bar is 50 μm

The disparity in the density of ADP-sensitive P2Y receptor subtypes in BM-MSCs among young vs. Pm women, prompted us to investigate their role in the osteogenic differentiation (ALP activity and bone nodule formation) using selective P2Y_1_, P2Y_12_ and P2Y_13_ antagonists, namely MRS 2179 (0.3 µM; *pKi* ∼ 7.0), AR-C66096 (0.1 µM; *pKi* ∼ 7.6), and MRS 2211 (10 µM; *pKi* ∼ 6.0), respectively [43] (Fig. 2).

The P2Y_1_ receptor antagonist, MRS 2179 (0.3 µM) reduced (*P* < 0.05) the ALP activity of BM-MSCs isolated from the younger female at culture day 28 (Fig. 2a). Contrariwise, blockage of P2Y_12_ and P2Y_13_ receptors with AR-C66096 (0.1 µM) and MRS 2211 (10 µM), respectively, favoured the osteogenic commitment of these cells (measured as increases in ALP activity) at the same time-point (Fig. 2a). Selective blockage of P2Y_1_, P2Y_12_ and P2Y_13_ receptor subtypes failed (*P* > 0.05) to modify BM-MSCs viability/proliferation (MTT assay; see Supplementary Fig. 5a) and the formation of mineralized bone nodules in cultures from the young pre-menopausal female (Fig. 2a).

On the contrary, selective blockage of P2Y_1_, P2Y_12_ or P2Y_13_ receptor subtypes had no measurable effects (*P* > 0.05) on the viability/proliferation (MTT assay; see Supplementary Fig. 5b), ALP activity and bone nodules formation by BM-MSCs from Pm women, when these cells were allowed to growth for 35 days in an osteogenic inducing medium (Fig. 2b). Thus, data suggest that endogenously formed ADP may have a moderate role in promoting osteogenic differentiation of BM-MSCs in younger pre-menopausal females, most probably via the activation of the P2Y_1_ receptor subtype. However, this effect is obliterated, at least in part, by tonic activation of co-localized P2Y_12_ and P2Y_13_ receptor subtypes. Previous studies demonstrated that overexpression of nucleotide metabolizing enzymes is responsible for the excessive breakdown and, thus, the loss of function of endogenously released adenine and uracil nucleotides by Pm BM-MSCs [15, 16]. This may explain the impairment of the osteogenic potential of these cells and the lack of P2Y_1_, P2Y_12_ or P2Y_13_ receptor tonus in this endeavour, providing that these receptors are not inactivated or desensitized.

### Activation of the P2Y_1_ receptor favours the osteogenic commitment of BM-MSCs from younger females, but not from Pm women, unless overexpressed P2Y_12_ and P2Y_13_ receptor subtypes are concurrently inhibited

**Fig. 3:**
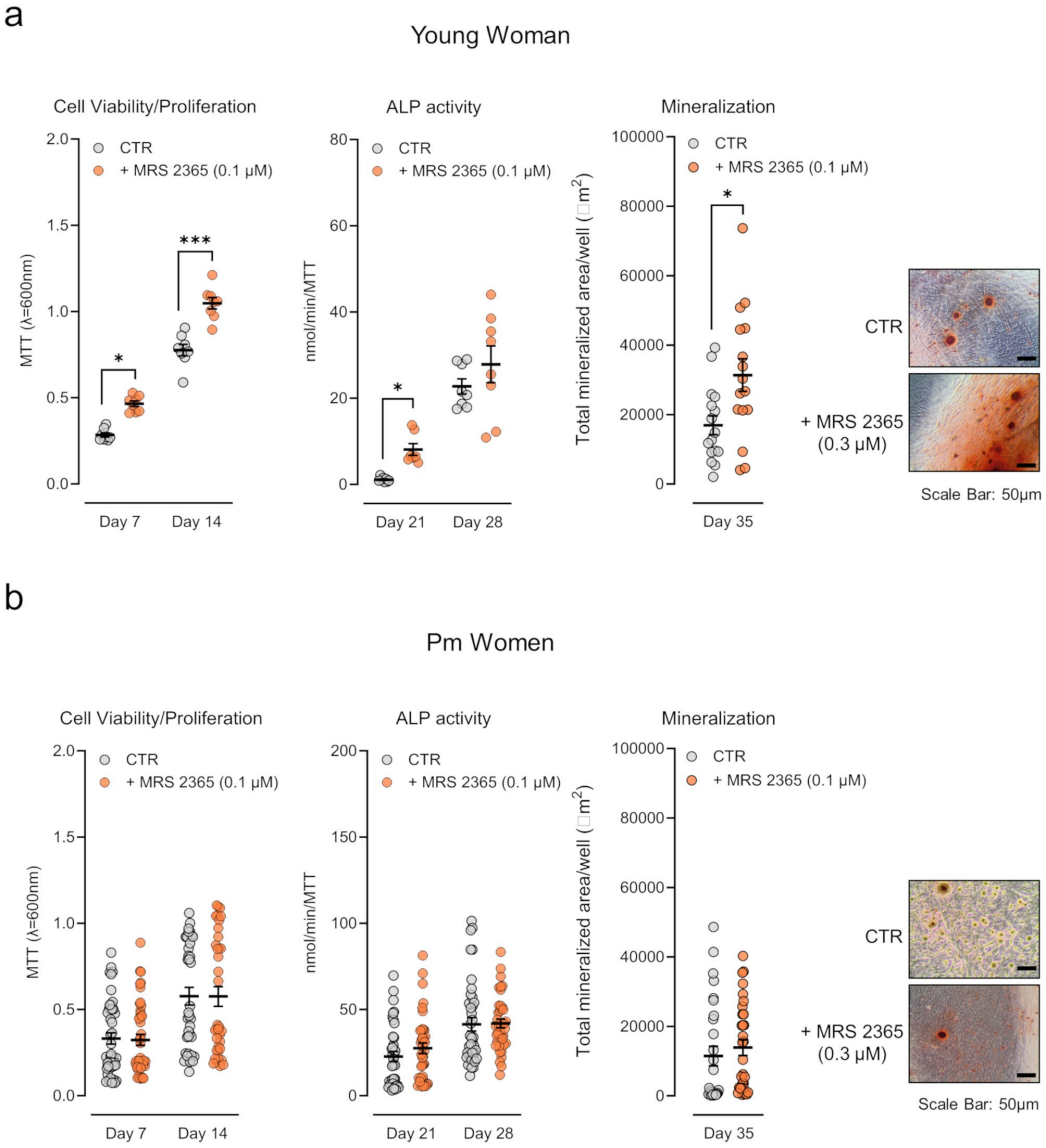
Selective activation of the P2Y_1_ receptor promotes BM-MSCs growth and osteogenic differentiation in a younger female, but not in Pm women. BM-MSCs were allowed to grow in an osteoblast-inducing medium for 7, 14, 21, 28, and 35 days, either in the absence or presence of the selective P2Y_1_ receptor agonist (MRS 2365, 0.1 µM). **Panels (a) and (b)** show, for (**a**) young and (**b**) Pm women BM-MSCs, the growth/viability (MTT assay), ALP activity (in nmol/min/MTT), and extracellular matrix mineralization (in µm^2^) for the indicated experimental groups. Scatter dot plots (with mean ± SD) represent pooled data from 1 young woman (37 years old) and 4 to 6 Pm women (72 ± 6 years old); four to sixteen replicates were performed per individual. *P < 0.05 and ***P < 0.001 (parametric ordinary one-way ANOVA with uncorrected Fisher’s LSD test and unpaired t-test for young woman results; non-parametric Kruskal-Wallis test with uncorrected Dunn’s test and two-tailed Mann Whitney test for Pm women) represent significant differences. Right hand-side images show typical micrographs displaying bone nodule formation (red-brownish spots) in BM-MSC cultures from a young female (37 years old) and a Pm woman (71 years old). Scale bar is 50 μm

The selective P2Y_1_ receptor agonist, MRS 2365 (0.1 µM), increased cells viability/proliferation (days 7 and 14), ALP activity (day 21) and mineralization of bone nodules (day 35) in primary cultures of BM-MSCs obtained from the 37-years old female (Fig. 3a). Yet, incubation with MRS 2365 (0.1 µM) failed to promote the osteogenic differentiation of BM-MSCs from Pm women cultured in similar experimental conditions (Fig. 3b).

It is worth noting that MRS 2365 selectively and potently activates the P2Y_1_ receptor subtype (EC_50_ = 0.4 nM) with almost no activity at P2Y_12_ and P2Y_13_ receptors, at least when used in submicromolar concentrations [43]. Yet, data show that (i) P2Y_12_ and P2Y_13_ receptors outnumber the P2Y_1_ receptor density in Pm BM-MSCs (see Fig. 1), and that (ii) activation of P2Y_12_ or P2Y_13_ receptor subtypes may negatively modulate the osteogenic effect of the P2Y_1_ receptor (see Fig. 2a). Given that these findings may confound MRS 2365 data interpretation, we set to investigate if selective blockage of overexpressed P2Y_12_ and P2Y_13_ receptors could re-establish the osteogenic action of the P2Y_1_ receptor in Pm BM-MSCs.

**Fig. 4:**
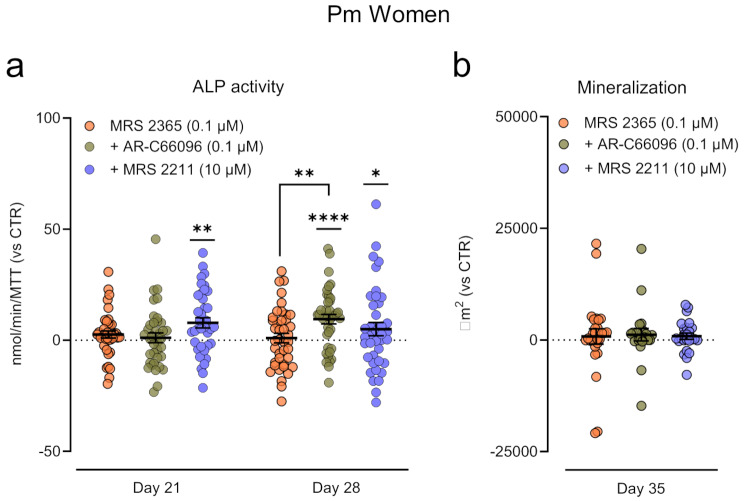
Blockage of P2Y_12_ and P2Y_13_ receptors partially rehabilitates the osteogenic potential of the P2Y_1_ receptor in Pm BM-MSCs. BM-MSCs were allowed to grow in an osteoblast-inducing medium for 21, 28, and 35 days, either in the presence of the selective P2Y_1_ receptor agonist (MRS 2365, 0.1 µM), applied alone or combined with the P2Y_12_ (AR-C66096, 0.1 µM) or P2Y_13_ (MRS 2211, 10 µM) selective antagonists. **Panels (a) and (b)** show the relative variation from the control situation (Δ = drug–CTR) in the (**a**) ALP activity (nmol/min/MTT) and (**b**) the extracellular matrix mineralization (µm^2^). Zero represents the identity between treated cells and ALP activity or total mineralized cell area obtained in non-treated (CTR) cells (horizontal dash line); control values are represented in Fig. 3b. Scatter dot plots (with mean ± SD) represent pooled data from 4 to 6 Pm women (72 ± 6 years old); four to eight replicates were performed per individual. *P < 0.05, **P < 0.01, and ****P < 0.0001 (non-parametric Kruskal-Wallis test with uncorrected Dunn’s test) represent significant differences. Right hand-side images show typical micrographs displaying bone nodule formation (red-brownish spots) in BM-MSC cultures from a Pm woman (71 years old). Scale bar is 50 μm

Figure 4a shows that selective blockage of P2Y_12_ or P2Y_13_ receptor subtypes with AR-C66096 (0.1 µM) and MRS 2211 (10 µM), respectively, partially re-admitted the osteogenic-inducing effect of MRS 2365 (0.1 µM), in BM-MSCs from Pm women, measured as increases in ALP activity at culture days 21 and 28. However, AR-C66096 (0.1 µM) and MRS 2211 (10 µM) were unable to promote the formation of bone nodules in Pm BM-MSCs challenged with the P2Y_1_ receptor agonist, MRS 2365 (0.1 µM; Fig. 4b), in contrast to that found with the cells of the younger pre-menopausal control (Fig. 3a).

### Transient P2Y_12_ or P2Y_13_ gene silencing using short hairpin RNAs also partially rehabilitates the P2Y_1_ receptor-induced osteogenic commitment of BM-MSCs from Pm women

Another possibility to avoid the negative impact of overexpressed P2Y_12_ and P2Y_13_ receptors in the osteogenic differentiation and matrix mineralization of Pm BM-MSCs is to promote specific gene silencing using short hairpin RNAs (shRNAs). To this end, we tested a series of lentiviruses carrying information to shRNAs designed to silence P2Y_12_ and P2Y_13_ gene expression. The most effective sequences were TL302719VA, at a multiplicity of infection 3 (MOI 3) against the P2Y_12_ receptor, and TL302718VD, at a multiplicity of infection 1 (MOI 1) against the P2Y_13_ receptor (ORIGENE, Rockville, MD, USA; RRID: SCR_008985). These sequences were selected among four blocking sequences (for each receptor) tested in parallel to a scramble sequence (negative control), which is unable to associate with the target mRNA (see Materials and Methods; validation experiments are shown in Supplementary Figs. 1 and 2).

**Fig. 5:**
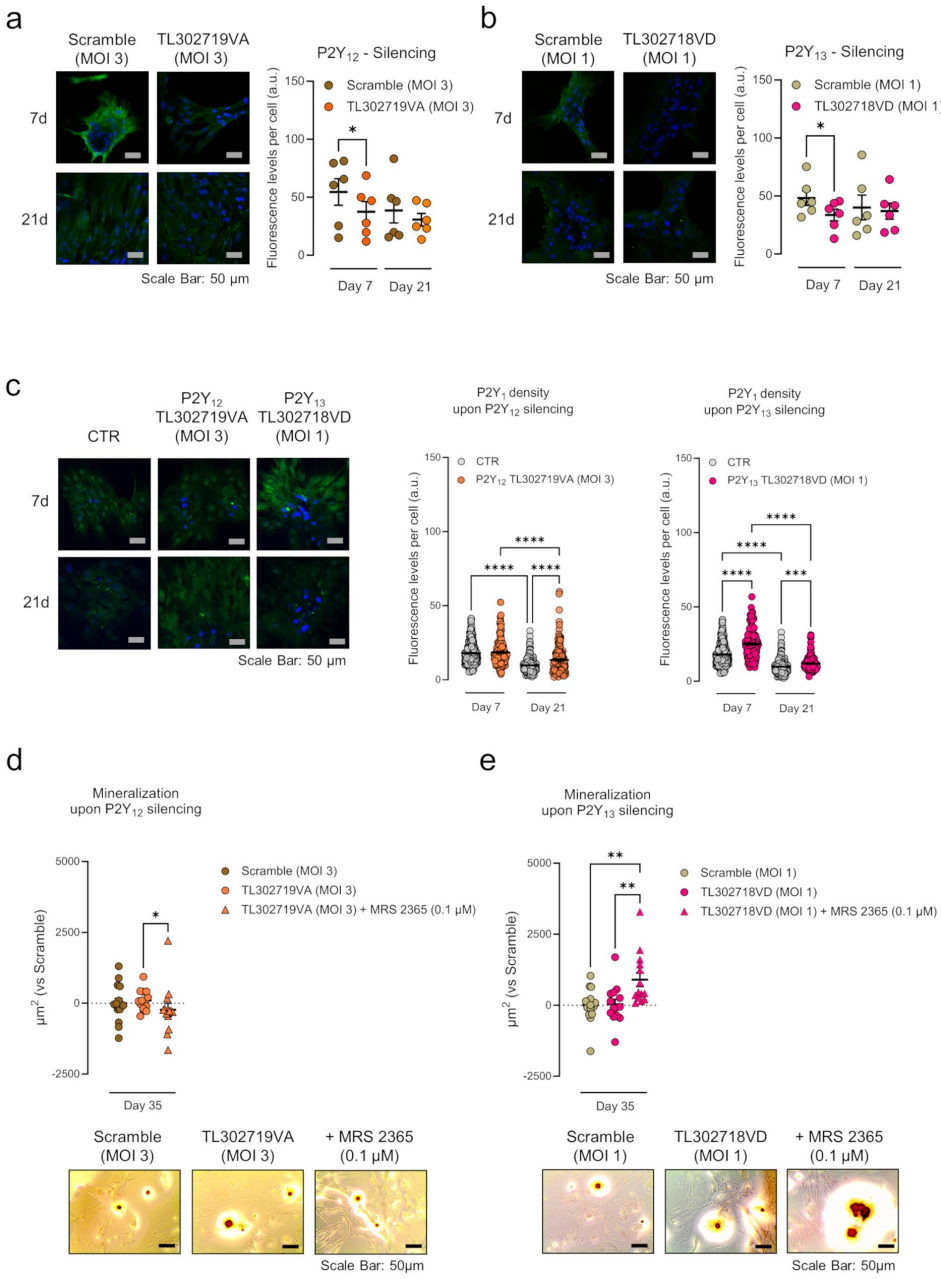
Transient P2Y_12_ or P2Y_13_ gene silencing using a short hairpin RNA partially rescues the P2Y_1_-mediated osteogenic differentiation in BM-MSCs from Pm women. Cells were exposed for 24 h to lenti-shRNAs designed to silence P2Y_12_ or P2Y_13_ receptors (or exposed to a scramble sequence) and then allowed to grow for 7, 21, and 35 days in an osteogenic-inducing medium. **Panels (a) and (b)** present representative immunofluorescence micrographs of Pm BM-MSCs from a Pm woman (83 years old) stained against (**a**) P2Y_12_ and (**b**) P2Y_13_ receptors (green). Blue dots represent nuclei stained with DAPI. The scale bar is 50 μm. In scatter dot plots (with mean ± SD), ordinates represent the fluorescence intensity per cell (arbitrary units, a.u.) of the indicated immunotarget as a function of the number of days in culture (day 7 and 21) from 6 Pm women (75 ± 6 years old) at the indicated experimental conditions. *P < 0.05 (RM one-way ANOVA with the Geisser-Greenhouse correction and uncorrected Fisher’s LSD test) represents significant differences. **Panel c)** presents representative immunofluorescence micrographs of BM-MSCs from a Pm woman (74 years old) stained against the P2Y_1_ receptor (green) after exposure to lenti-shRNA designed to silence the P2Y_12_ and P2Y_13_ receptors (day 7 and 21). Blue dots represent nuclei stained with DAPI. The scale bar is 50 μm. Shown are immunofluorescence intensity graphs computed from confocal microscopy images. Ordinates represent the fluorescence intensity per cell (arbitrary units, a.u.) of the P2Y_1_ receptor as a function of the number of days in culture (days 7 and 21) from a total of 147–374 cells analysed from 2 Pm women (73 ± 1 years old). ***P < 0.001 and ****P < 0.0001 (non-parametric Kruskal-Wallis with uncorrected Dunn’s test) represent significant differences. **Panel d) and e)** show the extracellular matrix mineralization (µm^2^) at culture day 35 after treatment with lenti-shRNA designed to silence (**d**) P2Y_12_ and (**e**) P2Y_13_ genes and exposed or not to P2Y_1_ selective agonist, MRS 2365 (0.1 µM). Zero represents the identity between represented conditions and the total mineralized cell area obtained in cells treated with the scramble sequence alone (horizontal dash line); differences between scramble sequence and non-treated cells (CTR) are represented in Supplementary Fig. 3. Scatter dot plots (with mean ± SD) represent pooled data from 4 Pm women (74 ± 6 years old); four to eight replicates were performed per individual. *P < 0.05 and **P < 0.01 (non-parametric Kruskal-Wallis test with uncorrected Dunn’s test) represent significant differences. Images at the bottom show typical micrographs displaying bone nodule formation (red-brownish spots) in BM-MSC cultures from a Pm woman (68 years old). Scale bar is 50 μm

Treatment of Pm BM-MSC cultures with the lenti-shRNAs encoding to sequences TL302719VA (MOI 3) and TL302718VD (MOI 1) decreased the P2Y_12_ and P2Y_13_ receptors immunoreactivity, respectively, when compared to the scramble sequence used at the same MOI, at culture day 7 (Fig. 5a and b). The P2Y_12_ and P2Y_13_ genes silencing was transient, given that differences against the scramble sequence were no longer observed at culture day 21 (Fig. 5a and b). Interestingly, the P2Y_12_ receptor gene silencing with the TL302719VA sequence (MOI 3) increased the relative density of the P2Y_1_ receptor subtype in Pm BM-MSCs, at culture day 21 (Fig. 5c). Concerning the effects operated by P2Y_13_ receptor gene silencing with the TL302718VD sequence (MOI 1), it consistently increased the P2Y_1_ receptor immunoreactivity in Pm BM-MSCs starting at culture day 7, which remained higher than the control until culture day 21 (Fig. 5c).

Despite the changes operated by selective silencing of P2Y_12_ and P2Y_13_ receptor genes in the P2Y_1_ receptor density in Pm BM-MSCs, these were not enough, on their own, to cause any impact on mineralization of the cultures, unless the cells were also challenged with the P2Y_1_ selective agonist, MRS 2365 (0.1 µM), for 35 days (Fig. 5d and e). This interaction was more evident when the silenced gene target was the P2Y_13_ receptor. It is worth noting that we detected no significant changes in the osteogenic commitment of BM-MSCs from Pm women when the scramble sequence was used either in the absence or presence of MRS 2365 (0.1 µM; see Supplementary Fig. 3c and 3d).

### Activation of the P2Y_1_ receptor failed to trigger [Ca^2+^]_I_ oscillations in BM-MSCs from Pm women unless cholesterol-rich lipid raft/caveola microdomains were disrupted to prevent lateral interference with overexpressed P2Y_13_ (and P2Y_12_) receptors

**Fig. 6:**
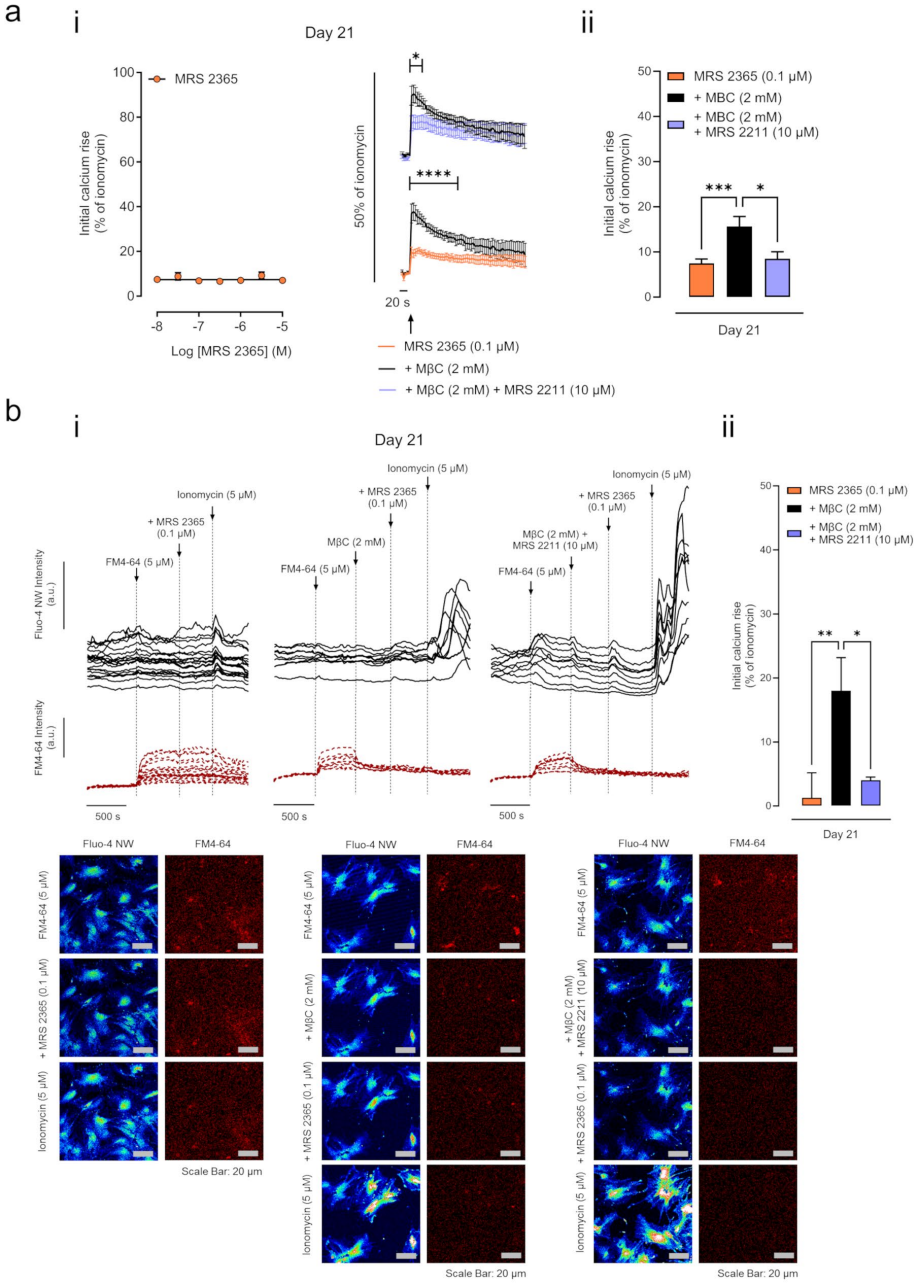
The P2Y_1_-induced [Ca^2+^]_i_ responses are compromised in Pm BM-MSC unless the negative interplay with overexpressed P2Y_13_ receptors in cholesterol-rich lipid raft/caveolae microdomains is abrogated with MβC. **Panel (a)** relates to microplate calcium measurements. **(a.i.)** Shows the concentration-response curve of [Ca^2+^]_i_ oscillations produced by MRS 2365 (0.01-10 µM) in Pm BM-MSCs allowed to grow for 21 days. The concentration-response curve was fitted using the least squares method by non-linear regression function: log(drug) versus response assuming that data share best-fit values from top and bottom and a hill slope equal to 1. Right hand-side graphs show fluorescence [Ca^2+^]_i_ transients in response to MRS 2365 (0.1 µM) either alone or in the presence of MβC (2 mM) with or without the selective P2Y_13_ receptor antagonist, MRS 2211 (10 µM). *P < 0.05 and ****P < 0.0001 (multiple unpaired t-tests) represent significant differences. **(aii)** Comparison of the magnitude of MRS 2365 (0.1 µM)-induces [Ca^2+^]_i_ rises in Pm BM-MSC cultures in the absence or presence of MβC (2 mM), either alone or applied with the selective P2Y_13_ receptor antagonist, MRS 2211 (10 µM). [Ca^2+^]_i_ transients were calibrated to the maximal calcium load produced by ionomycin (5 μm; 100% response). *P < 0.05 and ***P < 0.001 (non-parametric Kruskal-Wallis test with uncorrected Dunn’s test) represent significant differences. Bars and symbols (mean ± SD) represent pooled data from 2–9 Pm women (73 ± 5 years old); one to three replicates were performed per individual. **Panel (b)** Relates to single-cell [Ca^2+^]_i_ and FM4-64 fluorescence imaging in Pm BM-MSCs; the cells were previously loaded with the calcium-sensitive dye Fluo-4 NW (day culture 21), while incorporation of the FM4-64 styryl dye in the plasma membrane was assessed during perfusion of the cells on the stage of the confocal microscope (for details, see Materials and Methods). (**bi**) Shows representative traces of fluorescence oscillations induced by MRS 2365 (0.1 µM) applied either alone or in the presence of MβC (2 mM) with or without MRS 2211 (10 µM, selective P2Y_13_ receptor antagonist); the vertical lines indicate the time of drugs application. **(bii)** Comparison of the magnitude of MRS 2365 (0.1 µM)-induces [Ca^2+^]_i_ rises in Pm BM-MSC cultures in the absence or presence of MβC (2 mM), either alone or applied with the selective P2Y_13_ receptor antagonist, MRS 2211 (10 µM). Bars (mean ± SD) represent pooled data from a total of 8–18 cells analysed from 1 Pm woman (72 years old); *P < 0.05 and **P < 0.01 (ordinary one-way ANOVA with uncorrected Fisher’s LSD test) represent significant differences. Fluorescence confocal micrographs were obtained after the indicated drug application. The scale bar is 20 μm

Figure 6 shows that selective activation of the P2Y_1_ receptor with MRS 2365 (0.1 µM) failed to rise [Ca^2+^]_i_ inside BM-MSCs of Pm women undergoing osteogenic differentiation. This was assessed after loading the cells with the Ca^2+^ sensitive fluorescent dye, Fluo-4 NW, to measure [Ca^2+^]_i_ transients in cell population with a microplate reader (Fig. 6a) or single-cell [Ca^2+^]_I_ signals using the laser-scanning confocal microscope (Fig. 6b). Under such conditions, MRS 2365-induced intracellular [Ca^2+^]_i_ rises did not surpass 10% of the maximal Ca^2+^ load produced by ionomycin (5 µM) when the P2Y_1_ receptor agonist was used in the 0.01 to 10 µM concentration-range (Fig. 6a.i.).

The negative functional interplay between ADP-sensitive P2Y_1_, P2Y_12_ or P2Y_13_ receptor subtypes may depend on the crosstalk between metabotropic receptors, G proteins, and/or signalling transduction enzymes assembled in specialized plasma membrane microdomains highly enriched in cholesterol (lipid rafts, raft-like structures, caveolae) [reviewed in 44]. We addressed this question by testing the MRS 2365 (0.1 µM)-induced [Ca^2+^]_i_ oscillations inside Pm BM-MSCs in parallel with the synchronous incorporation of the FM4-64 fluorescent dye, which has been instrumental to evaluate membrane plasticity changes in living cells [41]. The impact of lipid raft/caveolae organization of the plasma membrane in the lateral interactions between overexpressed ADP-sensitive P2Y receptors was evaluated using methyl-β-cyclodextrin (MβC, 2 mM). This drug acutely depletes cholesterol from the plasma membrane of intact cells eventually resulting in the perturbation of lipid rafts/caveolae architecture [36, 42]. The red-stained micrographs in Fig. 6b show that depletion of membrane cholesterol with MβC (2 mM) causes a rapid decrease in FM4-64 fluorescent hotspots in the plasma membrane without causing any changes in [Ca^2+^]_i_ inside Pm BM-MSCs grown for 21 days in culture (see the time-lapse FM4-64 recording traces in Fig. 6b).

Pre-treatment of Pm BM-MSCs with MβC (2 mM) afforded the necessary conditions to unravel MRS 2365 (0.1 µM)-induced [Ca^2+^]_i_ rise (*P* < 0.05) in BM-MSCs of Pm women undergoing osteogenic differentiation (Fig. 6a and b), without modifying (*P* > 0.05) the FM4-64 fluorescent hotspots in the plasma membrane of these cells (Fig. 6b). Interestingly, the P2Y_1_ receptor-induced [Ca^2+^]_i_ transients observed after disruption of cholesterol-enriched membrane microdomains with MβC (2 mM) in Pm BM-MSCs were attenuated (*P* < 0.05) by the selective P2Y_13_ receptor antagonist, MRS 2211 (10 µM) (Fig. 6a and b). It is also worth noting that under similar experimental conditions, blockage of the P2Y_12_ receptor with AR-C66096 (0.1 µM) also attenuated (yet not significantly; *P* > 0.05) the MRS 2365 (0.1 µM)-induced [Ca^2+^]_i_ rises in Pm BM-MSCs after cholesterol depletion of plasma membrane microdomains with MβC (2 mM, Figure Supplementary 6a). Overall, data suggest that intracellular [Ca^2+^]_i_ oscillations operated by P2Y_1_ receptors activation in BM-MSCs from Pm women can only be detected once the negative interplay with overexpressed P2Y_13_ (and possibly P2Y_12_) receptors in cholesterol-rich lipid raft/caveolae microdomains is abrogated.

## Discussion

Using immunofluorescence confocal microscopy, we provide here compelling evidence that osteogenic-differentiating BM-MSCs originated from Pm women and a younger pre-menopausal control display variable amounts of ADP-sensitive P2Y_1_, P2Y_12_ and P2Y_13_ receptor proteins. In contrast to the findings obtained in younger female replicates, the density of the osteogenic-promoting P2Y_1_ receptor declined with time, whereas the amount of P2Y_12_ and P2Y_13_ receptors remained roughly conserved, in Pm BM-MSC primary cultures. This prompted us to investigate if uneven increases in the P2Y_12_/P2Y_1_ and P2Y_13_/P2Y_1_ receptor ratios could be responsible for failure of the osteogenic differentiation of Pm BM-MSCs induced by the P2Y_1_ receptor agonist compared to the cells obtained from the pre-menopausal control (see Figs. 3b, 4b and 6). Our main conclusion is that offsetting the activity and/or expression of P2Y_12_ or P2Y_13_ may be a good strategy to rehabilitate the loss of the osteogenic potential of P2Y_1_ receptor activation in Pm BM-MSCs (see e.g. Graphical Abstract). We reached this outcome using distinct experimental approaches, such as (i) selective receptor antagonists, (ii) receptors gene silencing with shRNAs, and (iii) blockage of lateral receptors interactions within lipid rafts/caveola microdomains upon depleting plasma membrane cholesterol, which all led us to comparable outcomes.

Our findings fully agree with data in the literature showing that osteoprogenitor cells from human bone explants exhibit different amounts of the ADP-sensitive P2Y_1_ receptor depending on donors’ age and differentiation stage of the cells in culture [20], which may negatively impact their osteogenic differentiation in Pm women (this study). While mRNA gene transcripts encoding the bone-promoting P2Y_1_ receptor were previously detected in human bone samples and osteoblast-like cell lines, namely MG-63, OHS-4 [45], SaOS-2 [46] and SaM-1 [47], the presence of the P2Y_12_ receptor was confirmed only in the osteosarcoma SaOS-2 cell line [46]. The presence of ADP-sensitive P2Y_1_, P2Y_12_ and P2Y_13_ receptors was demonstrated in primary osteoblast cell cultures of rat calvaria either at mRNA (RT-PCR and qPCR) and protein (flow cytometry and western blot analysis) levels [18, 21, 48–50], but, unlike rodents, there was no previous evidence for the existence of the P2Y_13_ receptor in human bone-forming cells, as we demonstrate here.

In contrast to the current knowledge that the P2Y_1_ receptor synergizes with PTH to increase bone formation [18, 19], the expression and activity of the P2Y_12_ receptor subtype in bone cells is still controversial. Prolonged application of clopidogrel, a pro-drug with P2Y_12_ receptor antagonist properties used as antiplatelet medication in humans, reduced growth, differentiation and bone nodule formation of rat osteoblast cultures with compatible in vivo repercussions in adult ovariectomized mice [21]. Surprisingly, these findings were still present in mice lacking the P2Y_12_ receptor [22]. Puzzling biphasic effects on the risk of fractures were also obtained in a cohort of Danish patients taking chronically clopidogrel to reduce heart attacks and stroke events [23]. Thus, one may interrogate about the mechanism of action of clopidogrel in bone turnover, given that this compound also inhibits Rho-associated kinase and inositol triphosphate/MAPK in platelets [51], which are normally associated to bone formation [14]. In this study, we used a highly selective P2Y_12_ receptor antagonist, AR-C66096, which on its own favoured the osteogenic differentiation (increases in ALP activity) of BM-MSCs from the 37-year old female, but not when the cells were isolated from Pm women. These results suggest that the P2Y_12_ receptor tone is downregulating the osteogenic commitment of BM-MSCs of younger females, but selective blockage of this receptor with compounds like AR-C66096 may unlock cells differentiation. The opposing effects between clopidogrel and AR-C66096 in bone osteoprogenitor cells growth and differentiation are not surprising taking into account that clopidogrel may be toxic to cells [21] in a concentration- and time-dependent manner [23].

Despite no studies have been performed so far focusing on the effects of the P2Y_13_ receptor in human bone-forming cells, osteoblasts originated from P2Y_13_ receptor knockout mice (P2Y_13_R^−/−^) have reduced differentiation capability and produce less bone nodules in vitro compared to the cells from wild-type animals. Downregulation of RhoA/ROCK 1 [24, 52] and downstream ERK-MAPK pathway in osteoblasts of animals lacking the P2Y_13_ receptor may explain their reduced osteogenic ability, which was also revealed by low Runx-2 protein levels and reduced ALP activity [53]. While BM-MSCs from wild animals respond to ADP with increases in ALP-colony-forming units (CFU-ALP) and expression of osteoblast-cell markers, like osterix, ALP and type I collagen, these effects were not observed in P2Y_13_R^−/−^ mice [26]. Interestingly, the osteogenic promoting effect of the P2Y_13_ receptor in mice may occur downstream of Runx-2, given that overexpression of this osteogenic marker was not affected in animals lacking the P2Y_13_ receptor [26]. In contrast to data obtained in mice, selective blockage of the P2Y_13_ receptor with MRS 2211 increased the ALP activity of BM-MSCs from the younger pre-menopausal female, but not when the cells were isolated from Pm women. These findings suggest that the osteogenic differentiation of BM-MSCs is offset by tonic activation of the P2Y_13_ receptor by endogenous ADP. Nevertheless, conflicting results may be attributed to differences in experimental conditions, target species, co-localized receptors’ interplay and/or downstream signalling crosstalk (see below).

Our data show that ADP-sensitive P2Y_12_ and P2Y_13_ receptor subtypes are overexpressed compared with the P2Y_1_ receptor density in immature/proliferating (7-day cultures) BM-MSCs isolated from Pm women, but this difference is tied in the younger control female, given that the density of P2Y_12_ and P2Y_13_ receptors increased 3 to 4-fold in differentiated 21-day BM-MSC cultures. Thus, normalization of P2Y_12_/P2Y_1_ and P2Y_13_/P2Y_1_ receptors density at later culture stages may not be enough to rehabilitate the loss of the P2Y_1_-mediated osteogenic commitment of Pm BM-MSCs *vis a vis* the younger control female, unless overexpressed P2Y_12_ and P2Y_13_ receptors interplay is counteracted early in the process of osteogenic differentiation. This strategy might be appropriate, since transient overexpression of the P2Y_1_ receptor early during the osteogenic differentiation of mesenchymal cells of the dental follicle (DFCs) may decrease matrix mineralization. The timely relevance of the P2Y_1_ receptor during the maturation of stem cells is highlighted by the fact that this receptor is downregulated in osteogenic-differentiated cells of the adipose tissue and dental follicle [54]. Here, we show that early blockage or activation of the P2Y_1_ receptor with MRS 2179 and MRS 2365, respectively, had no significant effects on growth (MTT assay), osteogenic differentiation (ALP activity) and formation of mineralized bone nodules by BM-MSCs from Pm women. These findings contrast with data obtained using BM-MSCs from the younger pre-menopausal female, where the P2Y_1_ receptor agonist, MRS 2365, increased growth (culture day 7 and 14), ALP activity (culture day 21) and mineralization of bone nodules (culture day 35), while the opposite was obtained concerning the ALP activity after blocking the P2Y_1_ receptor tone with MRS 2179. The lack of the P2Y_1_ receptor-mediated tone in Pm Bm-MSCs may be due (i) to insufficient extracellular ADP accumulation and/or (ii) to impairment of the P2Y_1_ osteogenic function resulting from the negative interplay with overexpressed P2Y_12_ and P2Y_13_ receptors, as discussed below.

A seminal work from our group showed that the endogenous actions of adenine and uracil nucleotides may be balanced through specific NTPDases determining whether human BM-MSCs are driven into proliferation or differentiation [15]. The immunoreactivity against NTPDase2, a preferential triphosphatase that is responsible for the breakdown of ATP yielding extracellular ADP accumulation, increases as BM-MSCs of Pm women differentiate; the vestigial immunoreactivity signal at culture day 7 becomes stronger at day 21 [15, 16], with almost no staining variations in the cells obtained from the younger female [16]. This may explain the lack of extracellular ADP accumulation at early cell differentiation stages and, therefore, the deficits in the osteogenic differentiation of Pm Bm-MSCs due to tonic activation of the P2Y_1_ receptor. A direct link between low NTPDase2 density and/or activity, overexpression of ADP-sensitive P2Y_12_ and P2Y_13_ receptors, and lack of the P2Y_1_ osteogenic tone in Pm BM-MSCS requires further investigations that are beyond the scope of this study. Our findings show that selective blockage of P2Y_12_ and P2Y_13_ receptors with AR-C66096 and MRS 2211, respectively, failed to affect the osteogenic differentiation of Pm BM-MSCs, but significantly enhanced the ALP activity in cells from the 37-years old woman. These findings strengthen our theory that extracellular ADP formation in Pm BM-MSC cultures do not reach enough levels to activate ADP-sensitive receptors.

We also hypothesized that impairment of the P2Y_1_-receptor-mediated osteogenic function in Pm BM-MSCs could also result from a negative influence provided by overexpressed P2Y_12_ and P2Y_13_ receptors at cholesterol-enriched plasma membrane microdomains. In fact, the selective blockage of the P2Y_12_ or P2Y_13_ receptors with pharmacological antagonists or these receptors’ gene silencing with lenti-shRNAs partially rehabilitated the osteogenic differentiation potential of the P2Y_1_ receptor agonist, MRS 2365, in Pm BM-MSC cultures ultimately leading to increases in the ALP activity and mineralization of bone nodules. It is worth noting that albeit P2Y_12_ and P2Y_13_ gene silencing curbed transiently the corresponding receptor proteins for only 7 days post-infection, this procedure was sufficient to rescue the P2Y_1_ receptor-mediated activity in BM-MSCs originated from Pm women. We obtained similar results using an analogous lenti-shRNA approach directed towards overexpression of the NTPDase3 enzyme to avoid excessive extracellular adenine and uracil nucleotides breakdown by Pm BM-MSCs [16]. Transient NTPDase3 gene silencing was also sufficient to increase the levels of the osteogenic transcription factor, osterix transcription, as well as the ALP activity and mineralization of bone nodules in Pm BM-MSC cultures to levels compared to those found using cells from younger females.

While pharmacological antagonists bind to specific receptor subtypes and compete with the natural ligand (e.g. ADP) in a concentration-dependent, gene silencing using the shRNA technology target a specific mRNA ultimately leading to a reduction in the synthesis of specific proteins. Looking in more detail at the results obtained using these two distinct approaches, some nuances may emerge. The pharmacological approach was more consistent considering the negative interplay between the P2Y_12_ receptor and readmission of the P2Y_1_-receptor-mediated osteogenic potential of Pm BM-MSCs detected as increases in the ALP activity (at culture day 28). Concerning the gene silencing approach, most evident results were obtained on matrix mineralization (at culture day 35) caused by the P2Y_1_-receptor agonist after Pm BM-MSCs infection with the lenti-shRNA preventing the P2Y_13_ receptor expression. Interestingly, gene silencing of P2Y_12_ and P2Y_13_ receptors both increased the density of the P2Y_1_-receptor in Pm BM-MSC, but this effect was particularly evident 7 days after cells infection with the lenti-shRNA designed to target the P2Y_13_ receptor gene transcript and, thus, contributing to decrease the P2Y_13_/P2Y_1_ density ratio.

The functional interplay between ADP-sensitive P2Y_1_, P2Y_12_ and P2Y_13_ receptors was previously reported in other cell systems. In astrocyte-microglial co-cultures, P2Y_1_ and P2Y_12_ receptors activation promotes astrocytes proliferation, whereas P2Y_12_ and P2Y_13_ receptors in microglia lead to the opposite effect [55]. Co-activation of P2Y_12_ receptors synergizes with the P2Y_1_ receptor to increase intracellular Ca^2+^ in human platelets [33] and rat glioma C6 cells [56]. These findings sustain the theory that the interplay between P2Y_1_, P2Y_12_ and P2Y_13_ receptors may have a significant impact on bone formation and remodelling, which we believe is particularly relevant in Pm women with high fracture risk. Most often, metabotropic receptors crosstalk occurs via lateral interaction and/or hetero-oligomerization at specific plasma membrane regions, like cholesterol-enriched lipid rafts and/or caveolae [reviewed in 44]. These specialized plasma membrane microdomains assemble most G protein-coupled receptors (like the ADP-sensitive P2Y_1_, P2Y_12_ and P2Y_13_ receptors), heterotrimeric G proteins, trafficking proteins, and key enzymes, such as kinases and phosphatases, which are responsible for extracellular signalling transduction, amplification and downstream activation of intracellular second messengers, ultimately affecting cells functioning [57].

Here, we show for the first time that cholesterol depletion with MβC resulted in significant disorganization of plasma membrane cell dynamics (revealed by the loss of FM4-64 fluorescent dye hotspots), which was sufficient to unravel previously unrecognized [Ca^2+^]_i_ transients caused by the P2Y_1_ receptor agonist, MRS 2365, in Pm BM-MSCs. The negative influence of overexpressed P2Y_13_ (and possibly P2Y_12_) receptors on P2Y_1_ receptor-induced [Ca^2+^]_i_ oscillations in this experimental setting was uncovered by blocking the former receptors with MRS 2211 (and AR-C66096), respectively, providing the acute disruption of cholesterol-rich lipid raft/caveolae microdomains with MβC. Interestingly, we observed this feature only in Pm BM-MSCs allowed to differentiate in culture for 21 days, but not in 7-day immature cells (data not shown), most probably because at a later time point normalization of unbalanced P2Y_12_/P2Y_1_ and P2Y_13_/P2Y_1_ receptor ratios is functionally more relevant. The P2Y_1_ receptor usually couples via a G_q/11_ anchor to the PLC/IP_3_/DAG pathway yielding intracellular [Ca^2+^]_i_ accumulation, whereas P2Y_12_ and P2Y_13_ receptors are more likely to couple to a G_i/o_ protein to inhibit the adenylate cyclase activity [27]. Despite no direct changes in [Ca^2+^]_i_ would be expected from P2Y_12_ and P2Y_13_ receptors activation, interactions of these receptors with other partners triggering intracellular [Ca^2+^]_i_ oscillations may affect cells differentiation and activity. For instance, blockage of G_i/o_ protein with pertussis toxin partly reduces the ability of ATP to increase [Ca^2+^]_i_ in adipose tissue-derived stem cells expressing P2Y_12_, P2Y_13_ and P2Y_14_ receptor subtypes [54]. Synergism to rise intracellular [Ca^2+^]_i_ has been observed between P2Y_1_ and PTH receptors; the latter is highly expressed in bone where it controls remodelling at discrete bone niches by coupling to both G_s_ and/or G_q_ protein families resulting in activation of adenylate cyclase and phospholipase C, respectively, depending on the hormone peptide concentration near bone-forming osteoblasts [58–60].

Complex interactions may also occur between distinct purinoceptors. The G_i/o_ protein-coupled adenosine A_1_ receptor may heterodimerize with the G_q_-coupled P2Y_1_ receptor either in heterologous transfected cells [61] and in rat brain tissues [62]. Heteromerization between A_1_ and P2Y_1_ receptors generates an adenosine receptor with a P2Y_1_-like agonist pharmacology; the potent P2Y_1_ receptor agonist, ADPβS, binds to the A_1_ receptor-binding pocket of the oligomer causing inhibition of the adenylyl cyclase activity via G_i/o_ protein, whereas binding of adenosine A_1_ receptor agonists and antagonists to the oligomer is significantly attenuated [61]. Adenosine A_1_ and P2Y_2_ receptors are also prone to oligomerize; synchronous activation of these two receptors induces a structural modification in the complex leading to inhibition of the A_1_ agonist binding and G_i/o_ signalling, while promoting increases in intracellular [Ca^2+^]_i_ via the G_q/11_-coupled P2Y_2_ purinoceptor [63]. Previously our group showed that selective activation of the adenosine A_1_ receptor subtype facilitates Pm BM-MSCs growth (MTT assay) and osteogenic differentiation (ALP activity) from the first culture week onwards; curiously, the localization of the A_1_ receptor at the plasma membrane decreases and internalization of this receptor becomes evident at later (28 days) culture stages [17]. Implications of A_1_/P2Y receptors oligomerization in the expression, localization and activity of both receptors concerning the deficits verified in osteogenesis and bone remodelling in Pm women certainly deserve thorough investigation in the future. Likewise, P2Y_1_ and P2Y_11_ receptors can also associate when co-transfected to HEK293 and 1321N1 astrocytoma cells; this interplay promotes agonist-induced internalization of the P2Y_11_ receptor, which on its own does not undergo endocytosis [64]. Thus, new insights about heteromeric receptor complexes provide the necessary molecular basis to explain the purinergic signalling diversity, while paving the way to investigate novel pathophysiological mechanisms and pharmacological drug targets. Considering the above information, it is tempting to speculate that personalized (pharmacological, genetic, environmental) manipulation of the expression, function and putative interplay between ADP-sensitive P2Y_1_, P2Y_12_ and P2Y_13_ receptors in BM-MSCs of Pm women and the younger control may re-equilibrate the role of these players to foster osteogenesis and bone formation in affected individuals. This attempt must not forget the possibility of fine-tuning modulate downstream receptor interactions at the second messengers’ level to rehabilitate the osteogenic potential of the P2Y_1_ receptor in Pm BM-MSCs.

Epigenetic and pharmacogenetic modifications induced by ageing, hormonal and/or environmental conditions may also influence ADP-sensitive P2Y_1_, P2Y_12_ and P2Y_13_ receptors activity in Pm BM-MSCs. In healthy volunteers (*n* = 200), the P2Y_1_ receptor gene exhibits five polymorphisms. The genetic dimorphism, 1622 A > G, has a significant clinical impact on platelet responses to ADP via the P2Y_1_, with greater responses found in subjects carrying the G allele. This genotype effect partly explains the inter-individual variation in platelet responses to ADP with significant repercussions in patients’ thrombotic risk and cardiovascular disease severity [65]. A putative role for six rare non-synonymous variants of the P2Y_1_ gene has been identified in a Polish population stratified for large-vessel ischemic stroke susceptibility [66]. However, controversy still exists on the influence of single nucleotide polymorphisms of the P2Y_1_ gene concerning the antiplatelet drug responsiveness of patients with coronary artery disease [67, 68]. Data from the present study encourage further investigations regarding putative variations of the P2Y_1_ receptor gene sequence and their functional implications in Pm women with high risk of fractures. These future prospects about the role of the P2Y_1_ receptor in human osteogenesis are strengthened by previous observations demonstrating that truncated splice variants of the P2X7 receptor in BM-MSCs might serve as fine descriptors of risk for bone mass loss and osteoporotic fractures (reviewed in [14]), besides the well-known implications of single-nucleotide polymorphisms of the P2X7 receptor gene [69].

This study presents a major limitation concerning the use of BM-MSCs from a single 37-year-old female due to scarcity of young female orthopaedic patients requiring bone engraftment for spinal instability/scoliosis correction (this study), high-impact trauma and/or pseudoarthrosis (or fracture mal-union) in our hospital. This failure, also considers that open hip surgeries are even less frequent in the younger than in aged population. Another issue could derive from distinct MSC phenotype as consequence of different sampling sources amongst young and Pm women, which is hardly proven considering available similar studies [14–16]. Despite these constrains, the observed differences by comparing the role of ADP-sensitive receptors in the osteogenic commitment of BM-MSCs from Pm women with that of the 37-year-old female, inspired our theory that unbalanced overexpression of P2Y_12_ and P2Y_13_ receptors in BM-MSCs may obscure the osteogenic effect of the P2Y_1_ receptor in aged Pm women, which fortunately could be demonstrated. These findings strengthen our previous results unravelling differences in the purinergic-signalling cascade of BM-MSC from young and Pm women cultured under similar experimental conditions, which have been focused on ATP-sensitive P2X7 [14] and UDP-sensitive P2Y_6_ receptors [15], mostly due to overexpression of the nucleotide-metabolizing enzyme, NTPDase3, on the plasma membrane of Pm BM-MSCs [16]. It is, however, worth noting that the main conclusions and the translational potential of the present study concerning individual differences in the interplay between ADP-sensitive receptor subtypes in BM-MSCs from Pm women are not affected by the aforementioned limitation, while the recruitment of younger patients certainly deserves attention in future studies.

## Conclusions

In this study, we show that osteogenic-differentiating Pm BM-MSCs display a different signature concerning ADP-sensitive P2Y_1_, P2Y_12_ and P2Y_13_ receptors compared to that found in a younger control female. Unbalanced overexpression of P2Y_12_ and P2Y_13_ receptors may at, least in part, explain the loss of the P2Y_1_-receptor-mediated osteogenic commitment of BM-MSCs in Pm women. Experimental data show that offsetting the activity and/or expression of P2Y_12_ and P2Y_13_ receptors, using (i) pharmacological inhibitors, (ii) gene silencing, or (iii) disturbance of cholesterol-enriched plasma membrane microdomains, may be a useful strategy to rehabilitate the osteogenic potential of the P2Y_1_ receptor in BM-MSCs from Pm women. The high heterogeneity in the expression and activity of the P2Y_1_ osteogenic promotor in Pm women encouraged us to propose a personalized therapeutic approach based on a biomarker-driven ideal proportion of this receptor *vis a vis* the P2Y_12_ and P2Y_13_ receptors to restore bone formation and remodelling in Pm women with high fracture risk.

## Electronic supplementary material

Below is the link to the electronic supplementary material.

Supplementary Figure 1: P2Y_12_ gene silencing (validation protocol) in BM-MSC cultures from a Pm woman. **Panel (a)** presents representative immunofluorescence micrographs of BM-MSCs from a Pm woman (83 years old) grown for 7 and 21 days in an osteogenic-inducing medium stained against P2Y_12_ receptors (green). Pm BM-MSCs were previously treated with several lenti-shRNAs encoding for four inhibitory and one scramble (negative control) sequences at increasing multiplicities of infection (MOI: 1, 3, 10). Blue dots represent nuclei stained with DAPI. The scale bar is 50μm. Table in **panel (b)** shows the percentage of the immunofluorescence levels obtained for the P2Y_12_ receptor for the indicated experimental protocols as compared to scramble (100%). Highlighted in green is the most effective sequence

Supplementary Figure 2: P2Y_13_ gene silencing (validation protocol) in BM-MSC cultures from a Pm woman. **Panel (a)** presents representative immunofluorescence micrographs of BM-MSCs from a Pm woman (83 years old) grown for 7 and 21 days in an osteogenic-inducing medium stained against P2Y_13_ receptors (green). Pm BM-MSCs were previously treated with several lenti-shRNAs encoding for four inhibitory and one scramble (negative control) sequences at increasing multiplicities of infection (MOI: 1, 3, 10). Blue dots represent nuclei stained with DAPI. The scale bar is 50μm. Table in **panel (b)** shows the percentage of the immunofluorescence levels obtained for the P2Y_12_ receptor for the indicated experimental protocols as compared to scramble (100%). Highlighted in green is the most effective sequence

Supplementary Figure 3: The scramble sequence did not affect the P2Y_12_ and P2Y_13_ receptors’ immunoreactivity, growth/viability and the osteogenic differentiation of BM-MSCs. In **panel a)**, ordinates represent the fluorescence intensity per cell (arbitrary units, a.u.) of the indicated immunotarget as a function of the number of days in culture (days 7 and 21). Scatter dot plots (with mean ± SD) represent pooled data from a total of 6 Pm women (75 ± 6 years old); Not significant (*ns*; RM one-way ANOVA with the Geisser-Greenhouse correction and uncorrected Fisher’s LSD test). **Panel b)** show the growth/viability (MTT assay) of Pm BM-MSCs grown for 7 and 21, previously treated (for 24h) with the scramble sequence or lenti-shRNAs designed to silence P2Y_12_ (TL302719VA MOI 3) or P2Y_13_ (TL302718VD MOI 1) receptors. Scatter dot plots (with mean ± SD) represent pooled data from 5 Pm women (75 ± 7 years old); four to eight replicates were performed per individual. Not significant (*ns*; non-parametric Kruskal-Wallis test with uncorrected Dunn’s test). **Panels c) and d)** show the ALP activity (nmol/min/MTT) and the extracellular matrix mineralization (µm^2^), respectively, of Pm BM-MSCs grown for 7, 21, and 35 days, previously treated (for 24h) with the scramble sequence and exposed or not to P2Y_1_ selective agonist, MRS 2365 (0.1 µM). Zero represents the identity between treated cells and ALP activity and the total mineralized cell area obtained in non-treated (CTR) cells (horizontal dashed line). Scatter dot plots (with mean ± SD) represent pooled data from 4 Pm women (74 ± 6 years old); four to eight replicates were performed per individual. Not significant (*ns*; non-parametric Kruskal-Wallis test with uncorrected Dunn’s test)

Supplementary Figure 4: Negative controls of immunofluorescence staining using Pm BM-MSCs grown for 21 days in an osteogenic-inducing medium. Shown are representative immunofluorescence micrographs of BM-MSCs from two Pm women (69 years for Anti rabbit Alexa Fluor 568 and 74 years for Anti rabbit Alexa Fluor 488), which were incubated with secondary antibodies (in the absence of primary antibodies) to detect non-specific fluorescence. Blue dots represent nuclei stained with DAPI. The scale bar is 50μm

Supplementary Figure 5: Selective blockage of P2Y_1_ (MRS 2179, 0.3 µM), P2Y_12_ (AR-C66096, 0.1 µM) and P2Y_13_ (MRS 2211, 10 µM) failed to affect growth/viability (MTT assay) of osteogenic-differentiating BM-MSCs isolated from young (**panel a**) and Pm (**panel b**) women in 28-day cultures. Scatter dot plots (with mean ± SD) represent pooled data from one 37 years-old control female and 3 to 5 Pm women (74 ± 5 years old); four to sixteen replicates were performed per individual. Non-parametric Kruskal-Wallis test with uncorrected Dunn’s test reveals no significant differences

Supplementary Figure 6: Disturbance of cholesterol-rich lipid raft/caveolae microdomains with MβC increases the magnitude of P2Y_1_-induced [Ca^2+^]_i_ transients in Pm BM-MSCs: on the role of P2Y_12_ receptor blockage. **Panel a)** shows MRS 2365 (0.1 µM)-induced [Ca^2+^]_i_ transients in a population of BM-MSCs from Pm women (microplate reader) either in the absence or presence of MβC (2 mM) applied alone or together with the selective P2Y_12_ receptor antagonist, AR-C66096 (0.1 µM). The right hand-side graph compares the magnitude of the fast [Ca^2+^]_i_ rise caused by indicated drugs. [Ca^2+^]_i_ transients were calibrated to the maximal calcium load produced by ionomycin (5μm; 100% response). ***P < 0.001 (non-parametric Kruskal-Wallis test with uncorrected Dunn’s test) represents significant differences. Bars (mean ± SD) represent pooled data from 4 to 9 Pm women (73 ± 5 years old); one to three replicates were performed per individual

## Data Availability

No datasets were generated or analysed during the current study.

## Abbreviations

A.u.: Arbitrary units
ADP: Adenosine 5′-diphosphate
Alizarin red S: 3,4-dihydroxy-9,10-dioxo-2-anthracenesulfonic
ALP: Alkaline phosphatase
AR-C66096: 2-(propylthio)adenosine-5’-O-(β,γ-difluoro methylene)triphosphate
ATP: Adenosine 5′-triphosphate
BM-MSCs: Bone marrow-derived mesenchymal stem cells
[Ca^2+^]_i_: Intracellular calcium
DFCs: Dental follicle cells
Lenti-shRNA: Lentiviral short hairpin RNA
MβC: Methyl-β-cyclodextrin
MOI: Multiplicity of infection
MRS 2179: 2’-Deoxy-N^6^-methyladenosine 3’,5’-bisphosphate
MRS 2211: 2-[(2-Chloro-5-nitrophenyl)azo]-5-hydroxy-6-methyl-3-[(phosphonooxy)methyl]-4-pyridinecarboxaldehyde
MRS 2365: [[(1R,2R,3 S,4R,5 S)-4-[6-Amino-2-(methylthio)-9 H-purin-9-yl]-2,3-dihydroxybicyclo[3.1.0]hex-1-yl]methyl] diphosphoric acid
MTT: 3-[4,5-dimethylthiazol-2-yl]-2,5-diphenyltetrasodium bromide
Pm: Post-menopausal
PNP: P-nitrophenyl phosphate
PTH: Parathyroid hormone
ROI: Regions of interest
