# Supplementary figures and images for: Silencing P2Y12 and P2Y13 receptors rehabilitates the ADP-induced P2Y1-mediated osteogenic commitment of post-menopausal mesenchymal stromal cells
Source: Cell Commun Signal. 2025 Jul 25;23:353. doi: 10.1186/s12964-025-02355-0 (PMC12291242; doi:10.1186/s12964-025-02355-0)

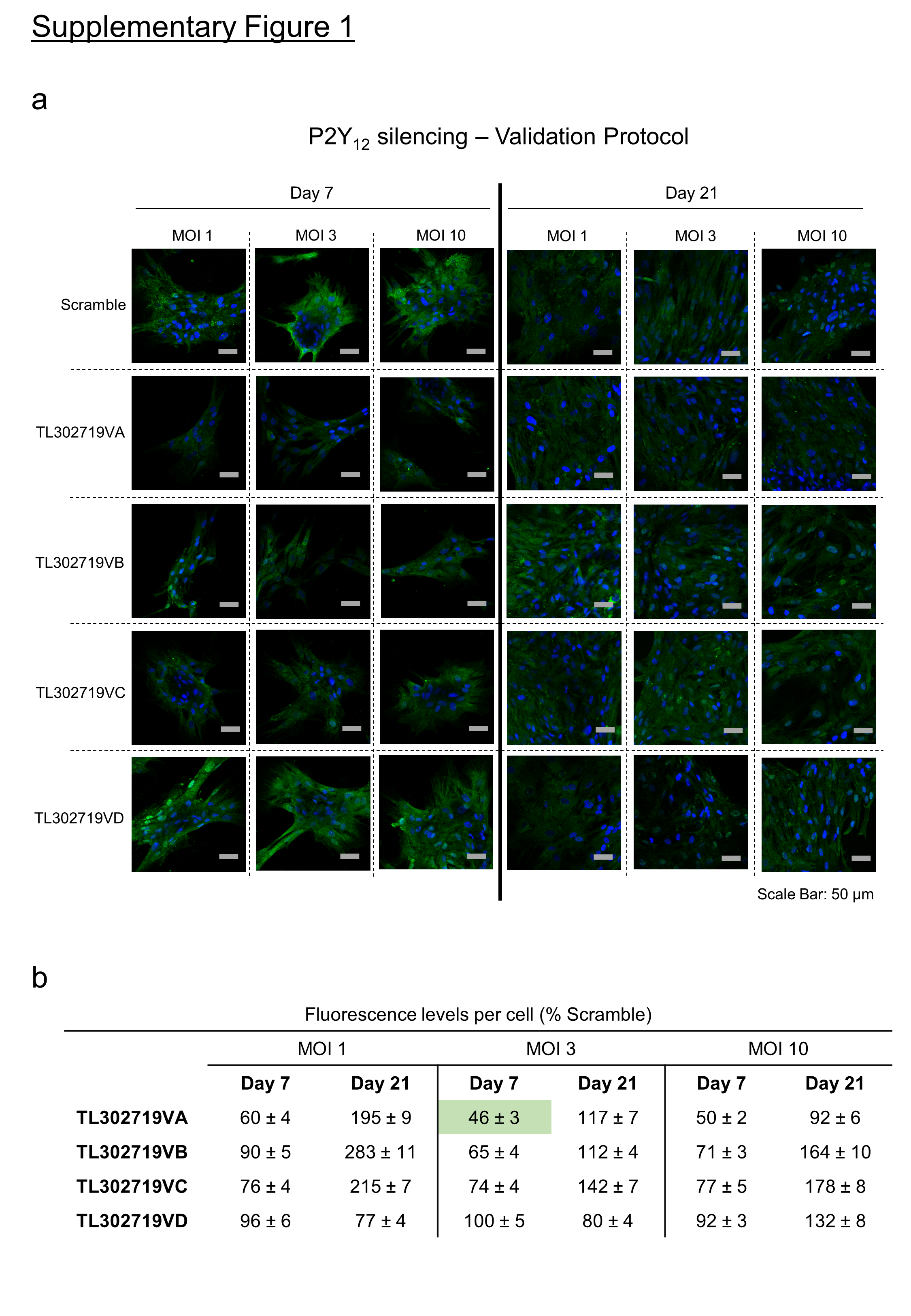

Supplement: Supplementary file 1 — Supplementary Figure 1: P2Y12 gene silencing (validation protocol) in BM-MSC cultures from a Pm woman. Panel (a) presents representative immunofluorescence micrographs of BM-MSCs from a Pm woman (83 years old) grown for 7 and 21 days in an osteogenic-inducing medium stained against P2Y12 receptors (green). Pm BM-MSCs were previously treated with several lenti-shRNAs encoding for four inhibitory and one scramble (negative control) sequences at increasing multiplicities of infection (MOI: 1, 3, 10). Blue dots represent nuclei stained with DAPI. The scale bar is 50μm. Table in panel (b) shows the percentage of the immunofluorescence levels obtained for the P2Y12 receptor for the indicated experimental protocols as compared to scramble (100%). Highlighted in green is the most effective sequence [file 12964_2025_2355_MOESM1_ESM.png]

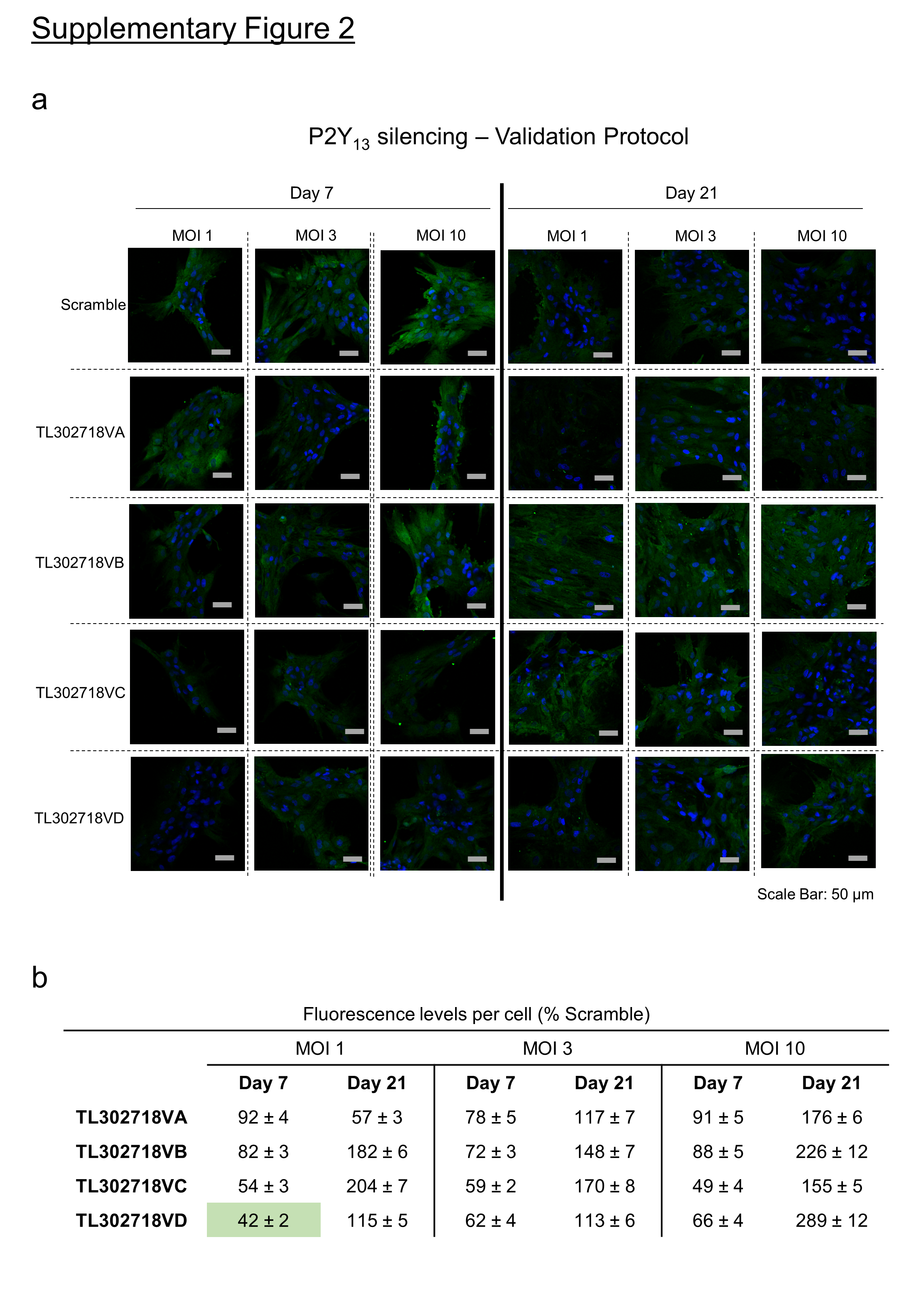

Supplement: Supplementary file 2 — Supplementary Figure 2: P2Y13 gene silencing (validation protocol) in BM-MSC cultures from a Pm woman. Panel (a) presents representative immunofluorescence micrographs of BM-MSCs from a Pm woman (83 years old) grown for 7 and 21 days in an osteogenic-inducing medium stained against P2Y13 receptors (green). Pm BM-MSCs were previously treated with several lenti-shRNAs encoding for four inhibitory and one scramble (negative control) sequences at increasing multiplicities of infection (MOI: 1, 3, 10). Blue dots represent nuclei stained with DAPI. The scale bar is 50μm. Table in panel (b) shows the percentage of the immunofluorescence levels obtained for the P2Y12 receptor for the indicated experimental protocols as compared to scramble (100%). Highlighted in green is the most effective sequence [file 12964_2025_2355_MOESM2_ESM.png]

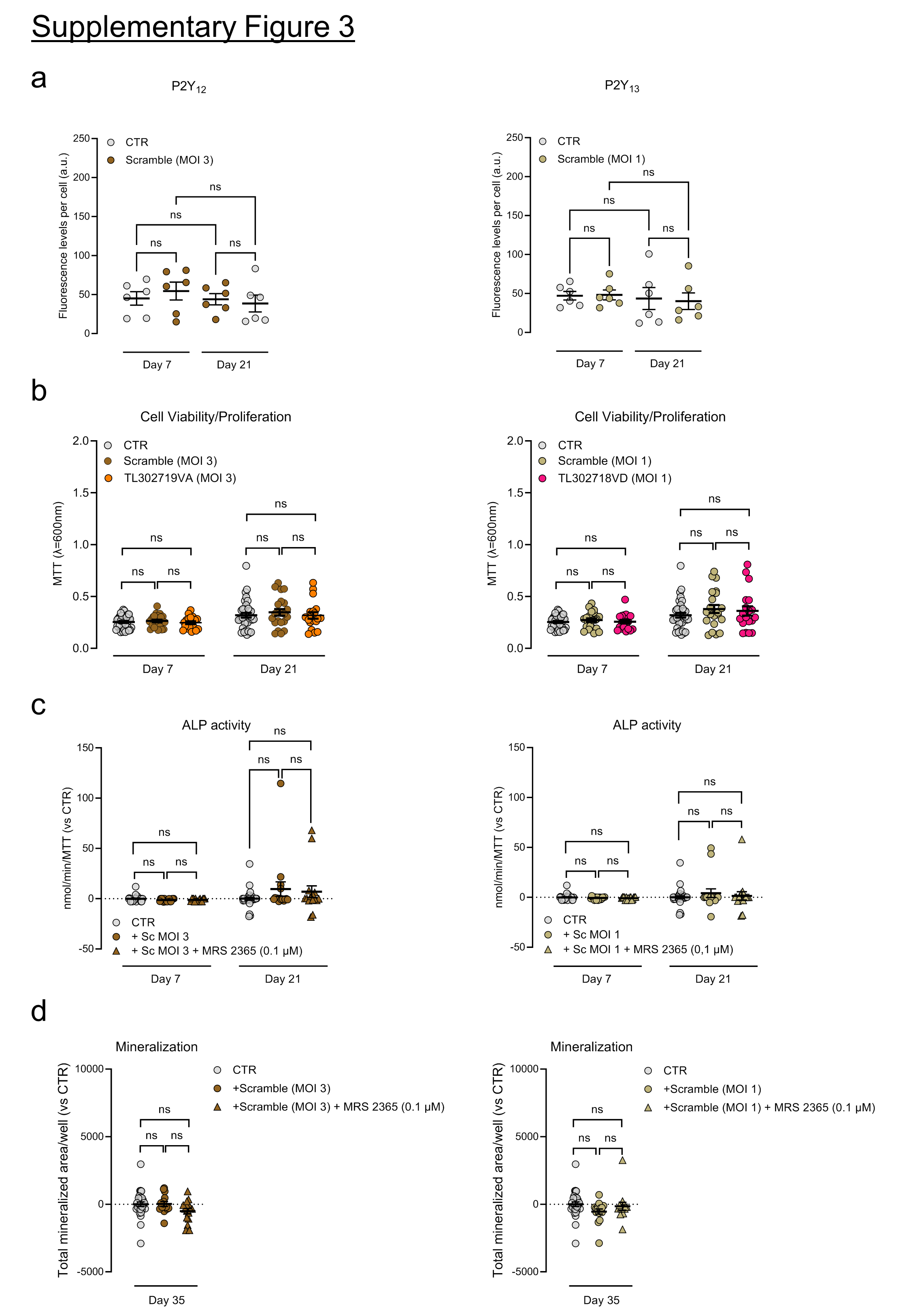

Supplement: Supplementary file 3 — Supplementary Figure 3: The scramble sequence did not affect the P2Y12 and P2Y13 receptors’ immunoreactivity, growth/viability and the osteogenic differentiation of BM-MSCs. In panel a), ordinates represent the fluorescence intensity per cell (arbitrary units, a.u.) of the indicated immunotarget as a function of the number of days in culture (days 7 and 21). Scatter dot plots (with mean ± SD) represent pooled data from a total of 6 Pm women (75 ± 6 years old); Not significant (ns; RM one-way ANOVA with the Geisser-Greenhouse correction and uncorrected Fisher’s LSD test). Panel b) show the growth/viability (MTT assay) of Pm BM-MSCs grown for 7 and 21, previously treated (for 24h) with the scramble sequence or lenti-shRNAs designed to silence P2Y12 (TL302719VA MOI 3) or P2Y13 (TL302718VD MOI 1) receptors. Scatter dot plots (with mean ± SD) represent pooled data from 5 Pm women (75 ± 7 years old); four to eight replicates were performed per individual. Not significant (ns; non-parametric Kruskal-Wallis test with uncorrected Dunn’s test). Panels c) and d) show the ALP activity (nmol/min/MTT) and the extracellular matrix mineralization (µm2), respectively, of Pm BM-MSCs grown for 7, 21, and 35 days, previously treated (for 24h) with the scramble sequence and exposed or not to P2Y1 selective agonist, MRS 2365 (0.1 µM). Zero represents the identity between treated cells and ALP activity and the total mineralized cell area obtained in non-treated (CTR) cells (horizontal dashed line). Scatter dot plots (with mean ± SD) represent pooled data from 4 Pm women (74 ± 6 years old); four to eight replicates were performed per individual. Not significant (ns; non-parametric Kruskal-Wallis test with uncorrected Dunn’s test) [file 12964_2025_2355_MOESM3_ESM.png]

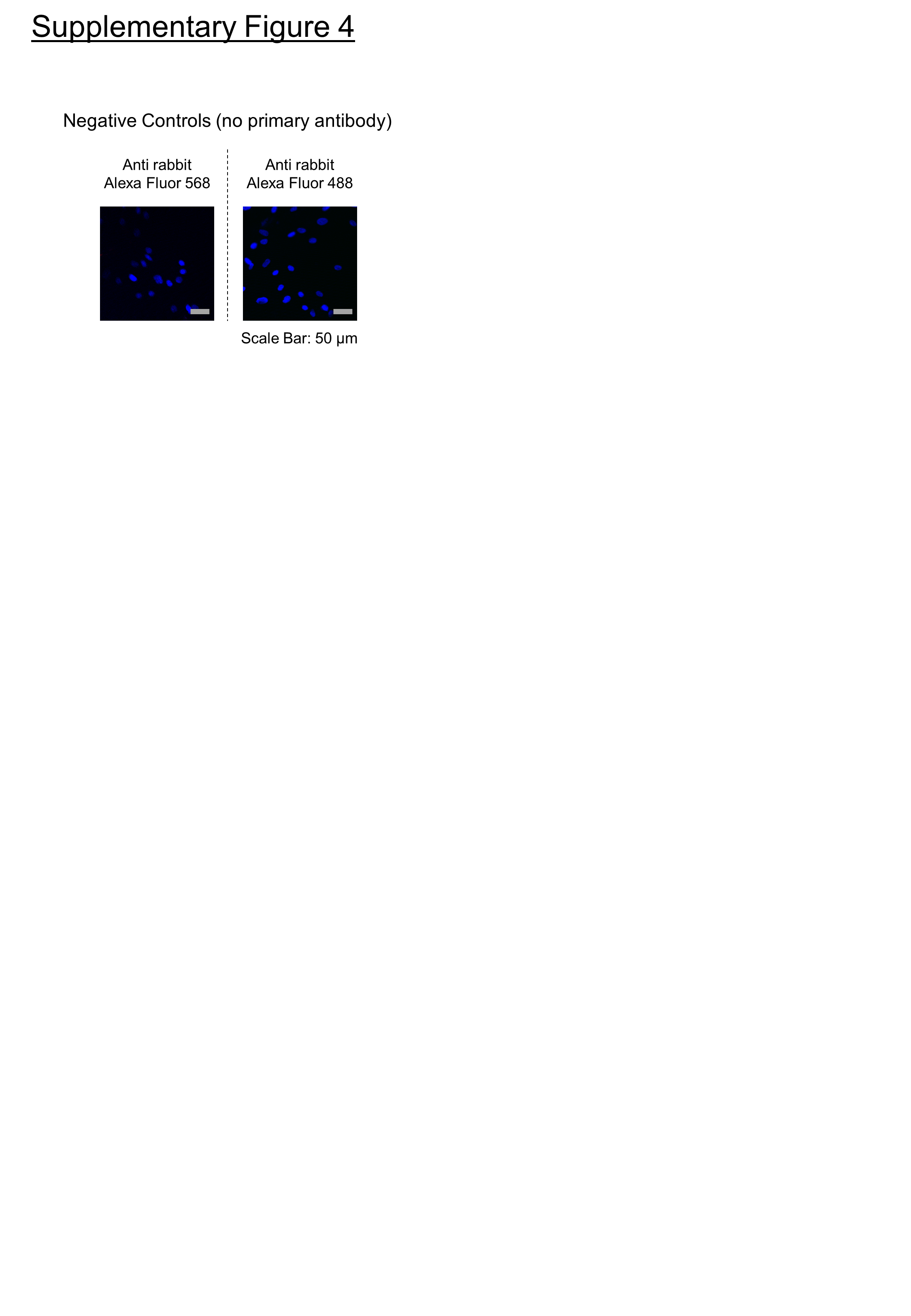

Supplement: Supplementary file 4 — Supplementary Figure 4: Negative controls of immunofluorescence staining using Pm BM-MSCs grown for 21 days in an osteogenic-inducing medium. Shown are representative immunofluorescence micrographs of BM-MSCs from two Pm women (69 years for Anti rabbit Alexa Fluor 568 and 74 years for Anti rabbit Alexa Fluor 488), which were incubated with secondary antibodies (in the absence of primary antibodies) to detect non-specific fluorescence. Blue dots represent nuclei stained with DAPI. The scale bar is 50μm [file 12964_2025_2355_MOESM4_ESM.png]

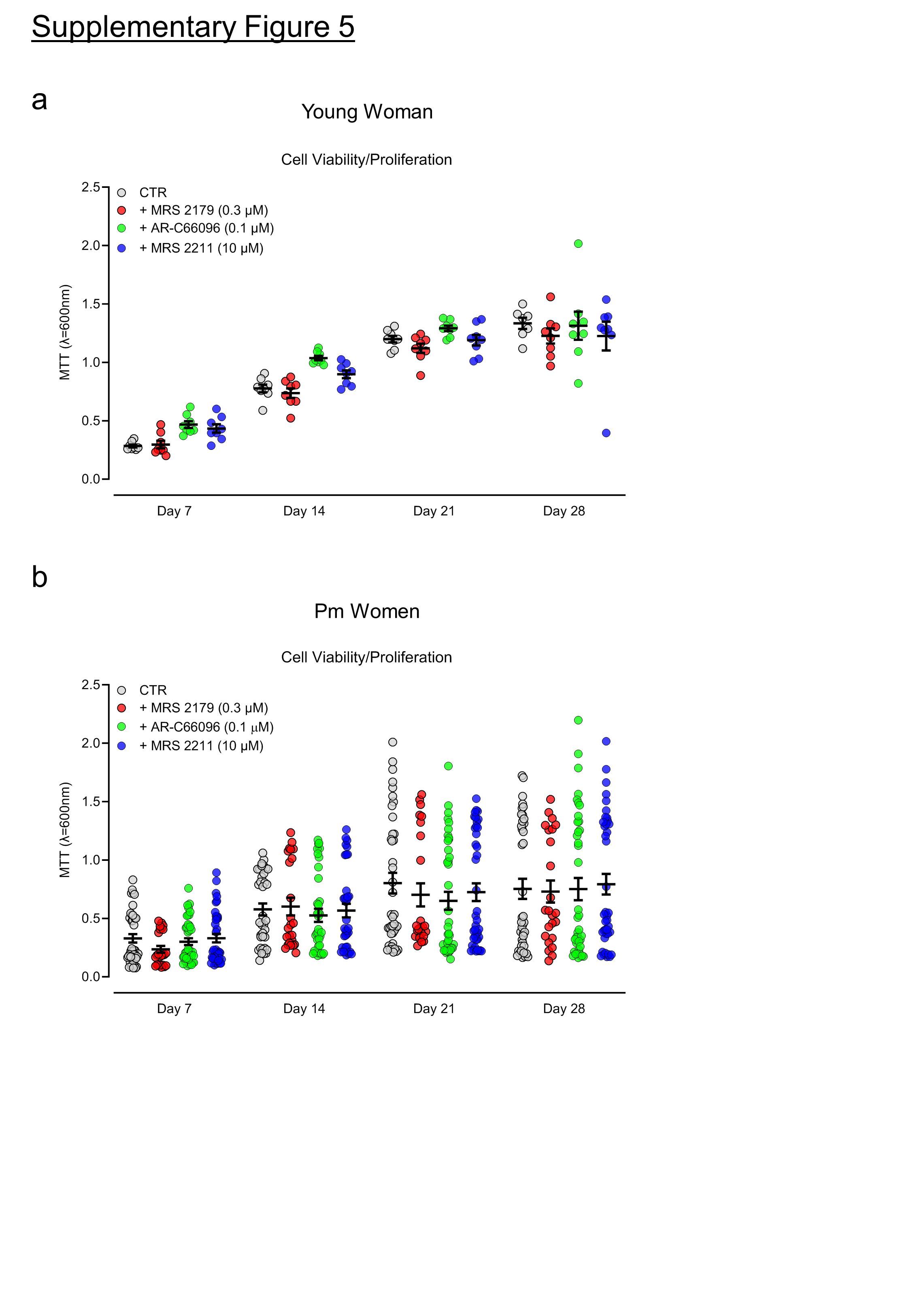

Supplement: Supplementary file 5 — Supplementary Figure 5: Selective blockage of P2Y1 (MRS 2179, 0.3 µM), P2Y12 (AR-C66096, 0.1 µM) and P2Y13 (MRS 2211, 10 µM) failed to affect growth/viability (MTT assay) of osteogenic-differentiating BM-MSCs isolated from young (panel a) and Pm (panel b) women in 28-day cultures. Scatter dot plots (with mean ± SD) represent pooled data from one 37 years-old control female and 3 to 5 Pm women (74 ± 5 years old); four to sixteen replicates were performed per individual. Non-parametric Kruskal-Wallis test with uncorrected Dunn’s test reveals no significant differences [file 12964_2025_2355_MOESM5_ESM.png]

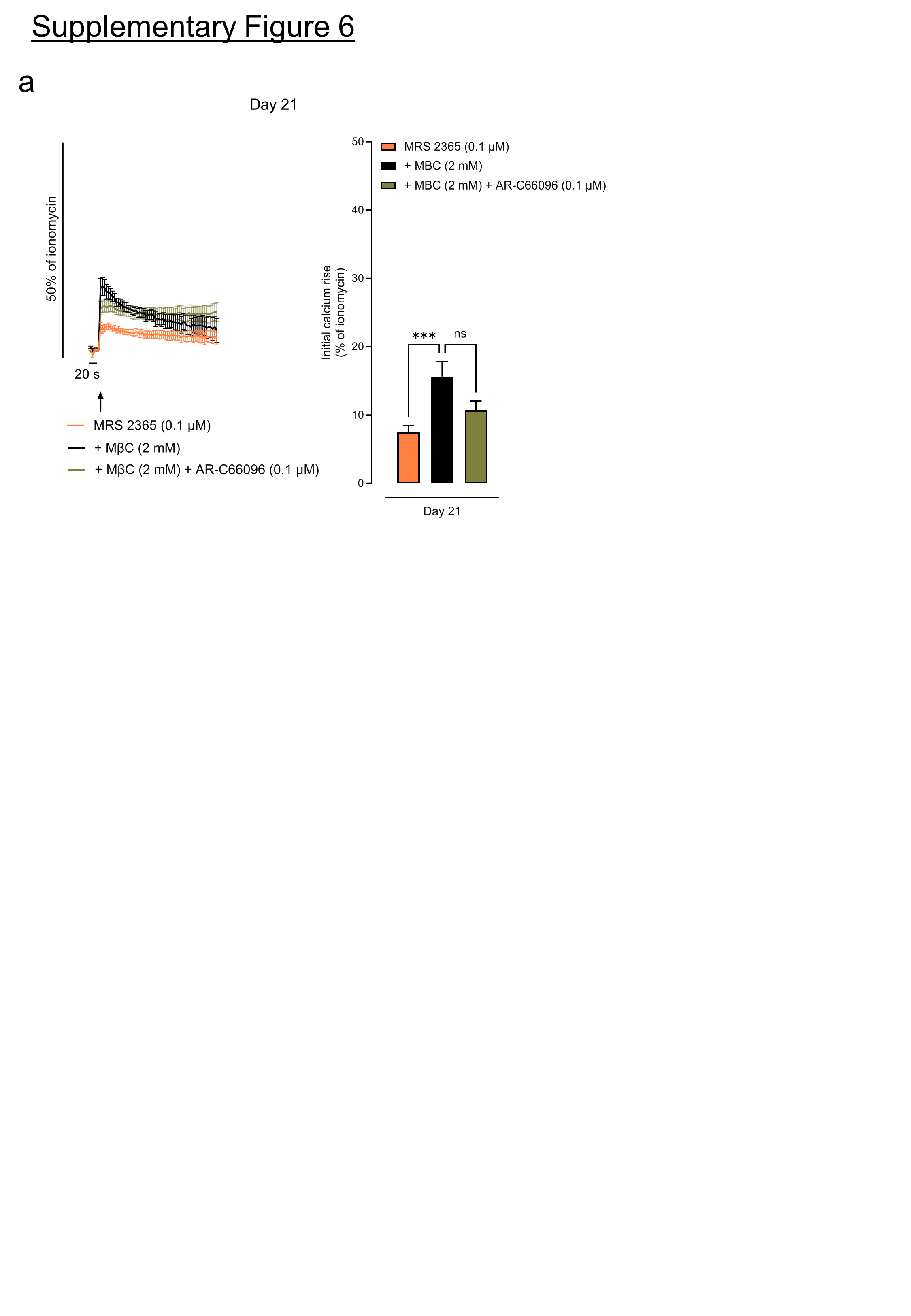

Supplement: Supplementary file 6 — Supplementary Figure 6: Disturbance of cholesterol-rich lipid raft/caveolae microdomains with MβC increases the magnitude of P2Y1-induced [Ca2+]i transients in Pm BM-MSCs: on the role of P2Y12 receptor blockage. Panel a) shows MRS 2365 (0.1 µM)-induced [Ca2+]i transients in a population of BM-MSCs from Pm women (microplate reader) either in the absence or presence of MβC (2 mM) applied alone or together with the selective P2Y12 receptor antagonist, AR-C66096 (0.1 µM). The right hand-side graph compares the magnitude of the fast [Ca2+]i rise caused by indicated drugs. [Ca2+]i transients were calibrated to the maximal calcium load produced by ionomycin (5μm; 100% response). ***P < 0.001 (non-parametric Kruskal-Wallis test with uncorrected Dunn’s test) represents significant differences. Bars (mean ± SD) represent pooled data from 4 to 9 Pm women (73 ± 5 years old); one to three replicates were performed per individual [file 12964_2025_2355_MOESM6_ESM.png]
